# The PhoPQ Two-Component System Is the Major Regulator of Cell Surface Properties, Stress Responses and Plant-Derived Substrate Utilisation During Development of *Pectobacterium versatile*-Host Plant Pathosystems

**DOI:** 10.3389/fmicb.2020.621391

**Published:** 2021-01-15

**Authors:** Uljana Kravchenko, Natalia Gogoleva, Nastassia Kalubaka, Alla Kruk, Yuliya Diubo, Yuri Gogolev, Yevgeny Nikolaichik

**Affiliations:** ^1^Department of Molecular Biology, Belarusian State University, Minsk, Belarus; ^2^Federal Research Center “Kazan Scientific Center of RAS”, Kazan Institute of Biochemistry and Biophysics, Kazan, Russia; ^3^Laboratory of Extreme Biology, Kazan Federal University Institute of Fundamental Medicine and Biology, Kazan, Russia; ^4^Department of Biochemistry, Biotechnology and Pharmacology, Kazan Federal University Institute of Fundamental Medicine and Biology, Kazan, Russia

**Keywords:** *Pectobacterium versatile*, PhoPQ, two-component system, pectin, arabinose, citrate, virulence

## Abstract

*Pectobacterium versatile* (formerly *P. carotovorum*) is a recently defined species of soft rot enterobacteria capable of infecting many plant hosts and damaging different tissues. Complex transcriptional regulation of virulence properties can be expected for such a versatile pathogen. However, the relevant information is available only for related species and is rather limited. The PhoPQ two-component system, originally described in pectobacteria as PehRS, was previously shown to regulate a single gene, *pehA*. Using an insertional *phoP* mutant of *Pectobacterium versatile* (earlier—*P. carotovorum*), we demonstrate that PhoP regulates at least 115 genes with a majority of them specific for pectobacteria. The functions performed by PhoP-controlled genes include degradation, transport and metabolism of plant-derived carbon sources (polygalacturonate, arabinose-containing polysaccharides and citrate), modification of bacterial cell envelope and stress resistance. We also demonstrated PhoP involvement in establishing the order of plant cell wall decomposition and utilisation of the corresponding breakdown products. Based on experimental data and *in silico* analysis, we defined a PhoP binding site motif and provided proof for its universality in enteric bacteria. Scanning *P. versatile* genome for the locations of this motif suggested a much larger PhoP regulon enriched with the genes important for a plant pathogen, which makes PhoP a global virulence regulator. Potential PhoP targets include many regulatory genes and PhoP control over one of them, *expI*, was confirmed experimentally, highlighting the link between the PhoPQ two-component and quorum sensing systems. High concentrations of calcium and magnesium ions were found to abolish the PhoPQ-dependent transcription activation but did not relieve repression. Reduced PhoP expression and minimisation of PhoP dependence of regulon members’ expression in *P. versatile* cells isolated from potato tuber tissues suggest that PhoPQ system is a key switch of expression levels of multiple virulence-related genes fine-tuned to control the development of *P. versatile*-host plant pathosystem.

## Introduction

*Pectobacterium* spp. are pectinolytic bacteria that cause soft rot and other diseases in a variety of plants. Until recently, members of this genus were often considered to be broad host range pathogens. However, genomic studies revealed significant diversity within this group, resulting in the ongoing subdivision of the previously described species and elevation of subspecies to species level. That resulted in better separation of the related strains according to their environmental preferences. *Candidatus Pectobacterium maceratum* was suggested as a new name for a group of strains isolated from potato and cabbage (Shirshikov et al., 2018). Recently, more isolates from various sources, including *Chrysanthemum*, *Iris* and water, were united with *Ca*. *P. maceratum* strains and renamed as *Pectobacterium versatile* (Portier et al., 2019). The *P. versatile* taxon includes two strains that have been well characterised previously, Ecc71 and SCC1. The 3-2 strain used in this work also belongs to this species.

*P. versatile* (*Pve*) strains can infect various plant hosts, and the same strain can cause diverse symptoms while infecting distinct tissues of the same plant (e.g., stem blackleg and tuber soft rot in potato) (Portier et al., 2019). Pectobacteria are also known to proliferate stealthily for a while in the vascular tissues before switching to a brute force attack of surrounding tissues via the massive secretion of plant cell wall hydrolases (Toth and Birch, 2005). Our previous work showed that initiation and development of the *Pectobacterium*-plant pathosystem requires extensive reprogramming of gene expression, both in the pathogen and its host (Gorshkov et al., 2018; Tsers et al., 2020). Such transcriptional reprogramming obviously involves multiple transcription factors. Yet, only a small fraction of about 300 transcription factors have been characterised in pectobacteria. These include pectinolysis and exoenzyme regulators KdgR (Liu et al., 1999), RexZ (Thomson et al., 1999), and GacA (Cui et al., 2001; Hyytiainen et al., 2001), the alternative sigma factor HrpL—the activator of the type III secretion system and its substrate genes (Chatterjee et al., 2002), motility regulators HexA (Harris et al., 1998), and FlhDC (Cui et al., 2008), pectin lyase regulators RdgA and RdgB (Liu et al., 1994) and quorum sensing regulators ExpR and VirR (Cui et al., 2005, 2006; Burr et al., 2006; Sjöblom et al., 2006).

PehR was originally described as a transcriptional activator of the *pehA* gene encoding endopolygalacturonase, a major virulence factor in soft rot bacteria (Flego et al., 2000). PehR is a response regulator that forms a two-component sensory system (TCS) with the membrane histidine kinase PehS. In the SCC3193 strain, which is currently classified as *Pectobacterium parmentieri*, inactivation of either *pehR* or *pehS* resulted in reduced virulence (Flego et al., 2000). The Ca^2+^ ion was reported as the PehS sensor ligand (Flego et al., 1997, 2000). It was also suggested that the PehRS system is responsible for the decrease of the polygalacturonase and increase of the pectate lyase activities in response to Ca^2+^ released from the degraded cell walls (Flego et al., 1997).

The pectobacterial PehRS system received very little attention since its discovery two decades ago. However, information has been accumulated about the orthologous PhoPQ TCS in other bacteria from the order *Enterobacterales*. In *Dickeya dadantii*, which belongs to the *Pectobacteriaceae* family together with *Pve*, PhoPQ was shown to control (Haque and Tsuyumu, 2005; Manjurul Haque et al., 2012) to cationic antimicrobial peptides (CAMPs) and expression of pectate lyases in response to changes of Mg^2+^ concentration (Haque and Tsuyumu, 2005; Manjurul Haque et al., 2012). The PhoPQ system has been thoroughly characterised in *Salmonella* and to a lesser extent in few more *Enterobacteriaceae* species including *Escherichia coli* and *Yersinia* spp. (see Groisman et al., 2013, for a review).

The PhoQ sensor histidine kinase activates PhoP by phosphorylation in response to several stimuli, including low Mg^2+^ concentration (Véscovi et al., 1996), cationic peptides (Bader et al., 2005), acidity (Prost et al., 2007) and high osmolarity (Yuan et al., 2017). PhoP binds to direct repeats with a consensus gGTTTA which seems to be well conserved in enterobacteria (Perez et al., 2009; Harari et al., 2010). The regulon composition nevertheless varies widely even between closely related bacteria. In *Salmonella enterica*, over a hundred genes are regulated by PhoP, but only three of them (*phoP*, *phoQ*, and *slyB*) are always under PhoP control in other enterobacteria. Some regulon members are shared by several species, but most PhoP controlled genes are species and even strain-specific (Perez et al., 2009). In summary, PhoP is a global regulator controlling diverse, but always large, regulons in *Enterobacterales*.

Despite the obvious importance of PhoP in *Enterobacterales*, its regulon has not been studied so far in *Pectobacterium* spp. To date, *pehA* remains the only known PhoP (PehR) target in these bacteria. The global mode of regulation reported for PhoP in other species strongly suggests that in pectobacteria it is likely to control many genes in addition to *pehA*. Moreover, in the recent work on *P. atrosepticum* transcriptome profiling *in planta*, we have noticed downregulation of *phoPQ* (Gorshkov et al., 2018). Expression level changes *in planta* combined with a potentially large regulon suggested that PhoP might be an important regulator of virulence properties in pectobacteria.

In this study we demonstrate that PhoP regulon of *Pve* includes at least 115 genes and incorporates a significant number of *Pectobacterium*-specific genes. We also try to distinguish the regulon parts directly and indirectly controlled by PhoP, discuss the implications of regulon composition for the regulation of *Pectobacterium* virulence and show an effect of divalent cations on the PhoP/PhoQ TCS.

## Materials and Methods

### Bacterial Strains and Growth Conditions

*Pve* strain JN42 (Myamin et al., 2004) is a spontaneous rifampicin-resistant derivative of the wild type isolate 3-2, originally described as *Erwinia carotovora*. The *Escherichia coli* strain XL-1 Blue (Bullock, 1987) was primarily used for plasmid construction and *E. coli* strain BW 19851 (Metcalf et al., 1994) was used for conjugational transfer of suicide vector pJP5603 (Penfold and Pemberton, 1992) derivatives into *Pve*. *Pve* and *E. coli* were routinely grown in lysogeny broth (LB) or minimal media at 28 and 37°C, respectively. For *N-*acyl-homoserine lactone bioassay, *Chromobacterium violaceum* CV026 was grown as described (McClean et al., 1997).

Two minimal media were used throughout this work. Minimal medium A (MMA) was composed of K_2_HPO_4_ (10.5 g/l), KH_2_PO_4_ (4.5 g/l), (NH_4_)_2_SO_4_ (1 g/l), sodium citrate (0.6 g/l), and 0.2% glycerol. MgSO_4_ was added to MMA to the final concentration of 0.5 mM. Sodium polypectate (Sigma) or L-arabinose were added to the final concentrations of 0.5 and 0.2% when necessary. To avoid precipitation, the effects of different divalent cation concentrations were studied in minimal medium N (MMN) (Nelson and Kennedy, 1971) containing KCl (5 mM), (NH_4_)_2_S0_4_ (7.5 mM), K_2_S0_4_ (0.5 mM), KH_2_PO_4_ (1 mM), Tris-HCl (0.1 M), pH 7.4, and 0.2% glycerol. MgSO_4_ and CaCl_2_ were added to MMN to either 10 μM or 10 mM as required.

Antibiotics were used at the following concentrations (μg/ml): ampicillin, 100; gentamycin, 10; rifampicin, 25; kanamycin, 20.

### Construction of the *phoP* Mutant and Complementation Plasmid

To construct a *phoP* mutant of *Pve*, the *phoP* gene sequence was PCR amplified from *Pve* 3-2 with phoPf and phoPr primers (Supplementary Table 1) and its internal *Nde*I-*Pvu*II fragment (225 bp) was cloned into the suicide vector pJP5603. The resulting plasmid was mobilised into *Pve* from *E. coli* BW 19851 and *Pve* crossover clones were selected on kanamycin (20 μg/ml) containing plates. *Pve* disruption was confirmed by PCR with combinations of primers to *phoP* and suicide vector sequences (phoPf-phoPr, phoPf-pjp2, and phoPr-pjp1, Supplementary Table 1).

The plasmid expressing *phoP* and *phoQ* was constructed by cloning the PCR fragment amplified with the Tersus DNA polymerase (Evrogen) and the phoPQf1 and phoPQr primers into the low copy number vector pZH449.

Characteristics of the plasmids used in this work are specified in Supplementary Table 2.

### RNA-Seq Analysis

For RNA preparation from *Pve* 3-2 or *phoP*-mutant cultures, cells were aerobically grown at 28°C in MMA with sodium polypectate to an optical density at 600 nm (OD600) of 0.4. The details of RNA isolation, cDNA library preparation, sequencing and general characteristics of the RNA-seq data generated have been described (Gogoleva et al., 2020). 84.2 million reads were generated in total with four biological replicates sequenced for each type of the libraries (wild type and *phoP* mutant). The obtained read sequences corresponding to the *Pve* genome can be accessed from NCBI’s BioProject under the accession number PRJNA627079.

For quantification of gene expression levels, the coding sequences of *Pve* 3-2 genome were used as a reference (GenBank accession CP024842). Read pseudo-alignment and transcript quantification was performed using the alignment-free kallisto tool (Bray et al., 2016). The analysis of the differentially expressed genes (DEGs) was carried out with the edgeR package (Robinson et al., 2010). Genes with fold-change ≥ 2 and significant differences in expression levels (FDR < 0.05) were considered as DEGs.

### Gene Expression Analysis by qRT-PCR

The total RNA from *Pve* cells was extracted, treated with DNAse and quantified as described above. One microgram of RNA was used for cDNA synthesis using RevertAid reverse transcriptase (Thermo Fisher Scientific) according to the manufacturer’s instructions. Two microliter of fivefold-diluted cDNA were used as the template for qPCR.

qPCR was performed using a SYBR Green I—containing master mix. Primers for target and reference genes (Supplementary Table 1) were designed using Primer3 software (Untergasser et al., 2012) and checked for specificity with Primer-Blast (Ye et al., 2012). PCR was performed under the following conditions: 95°C for 2 min, followed by 45 cycles at 94°C for 10 s and 60°C for 60 s. After that, melting curve analysis was performed in the temperature range of 60–90°C. The reactions were run and changes in fluorescence emission were detected using a DT-96 quantitative PCR system (DNA Technology, Russia). The amount of fluorescence was plotted as a function of the PCR cycle and converted to Ct values using RealTime_PCR Software (DNA Technology, Russia). The amplification efficiency for all primers was determined using a dilution series of genomic DNA. Additional controls included the omission of reverse transcriptase to measure the extent of residual genomic DNA contamination and template omission. The *ffh* and *gyrA* genes, the transcript levels of which were confirmed by the geNorm software (Vandesompele et al., 2002) to be stable under the applied experimental conditions (data not shown), were used for normalisation of the expression of the target genes. At least four biological replicates were performed for each measurement. Relative expression levels and error estimates were calculated using the REST 2009 v. 2.0.13 software (Pfaffl et al., 2002).

### Transcription Factor Binding Site Inference and Correction of Genome Annotation

ChIPmunk (Kulakovskiy et al., 2010) was used for the inference of PhoP binding sites within the regulatory regions of the differentially expressed genes. The ChIPhorde version of the algorithm was run, followed by manual filtering of putative operators according to their scores and positions relative to transcription initiation sites. TFBS (Transcription factor binding site) positions in the context of RNA-seq coverage were visualised by SigmoID (Nikolaichik and Damienikan, 2016). Wig files with RNA-seq coverage data were generated by Rockhopper (McClure et al., 2013).

Inference of operator motifs for other TFs was done with the modified algorithm of Sahota and Stormo (2010) as implemented in SigmoID version 2.0^1^. SigmoID was also used for scanning the *Pve* genome for operator sequences matching known or new motifs. Positions of critical residues required by this algorithm were determined by the interaction service of NPIDB (Zanegina et al., 2016). Alignment of the DNA binding domains was done with hmmalign from the HMMER3 package (Eddy, 2011).

Correction of annotation for PhoP controlled genes was done using search and editing capabilities of SigmoID (Nikolaichik and Damienikan, 2016). The updated annotation of the *Pve* 3-2 genome was submitted to GenBank under the same accession number.

### Virulence Assays

Bacteria were grown overnight on solid LB plates, washed off with 0.85% NaCl solution, centrifuged briefly and resuspended in the same solution, after which cell suspension densities were adjusted to achieve the cell doses 5⋅10^5^ (high inoculum dose) or 1⋅10^5^ (low inoculum dose) per inoculation site. Potato (*Solanum tuberosum*) cv Krone grade E basic seed tubers were obtained from a local seed potato producer. The tubers were inoculated with 10 μl of cell suspensions using a 200 μl pipette tip close to stolon end of the tuber. The inoculation sites were wrapped in parafilm and tubers were kept in plastic bags for 48 h at 28°C.

Inoculum doses were chosen in preliminary experiments as follows. Minimal cell numbers sufficient for reliable disease development by the wild type strain were taken as low inoculum dose. Higher cell numbers that could stably produce different test results were taken as high inoculum dose.

For Chinese cabbage inoculation, cell suspensions were prepared the same way, but their cell densities were adjusted to the values 1⋅10^5^ (high inoculum dose) or 1⋅10^3^ (low inoculum dose) per inoculation site. Chinese cabbage leaves were soaked in the sodium hypochlorite solution (70–85 g/l of active chlorine) and then flushed with sterile deionised water. Rectangular sections were cut out from the base of each leaf. The obtained sections were soaked in the 96% ethanol and then flamed. The sections were inoculated with 10 μl of cell suspensions using a 200 μl pipette tip. The samples were placed in the sterile Petri dishes and incubated for 48 h at 28°C.

At least 10 tubers or leaf sections were used per each experimental condition. 0.85% NaCl solution was used for control inoculations.

## Results

### Inactivation of *phoP* Alters *P. versatile* Virulence

To examine the role of PhoP in *P. versatile* interaction with host plants, we compared the properties of the *phoP* mutant strain with its wild type parent and a complementation strain. Since *phoP* is the first gene of the *phoPQ* operon and a polar effect of *phoP* inactivation on the expression of *phoQ* was expected, complementation was achieved by a plasmid carrying the whole *phoPQ* operon together with the full regulatory region.

**FIGURE 1:**
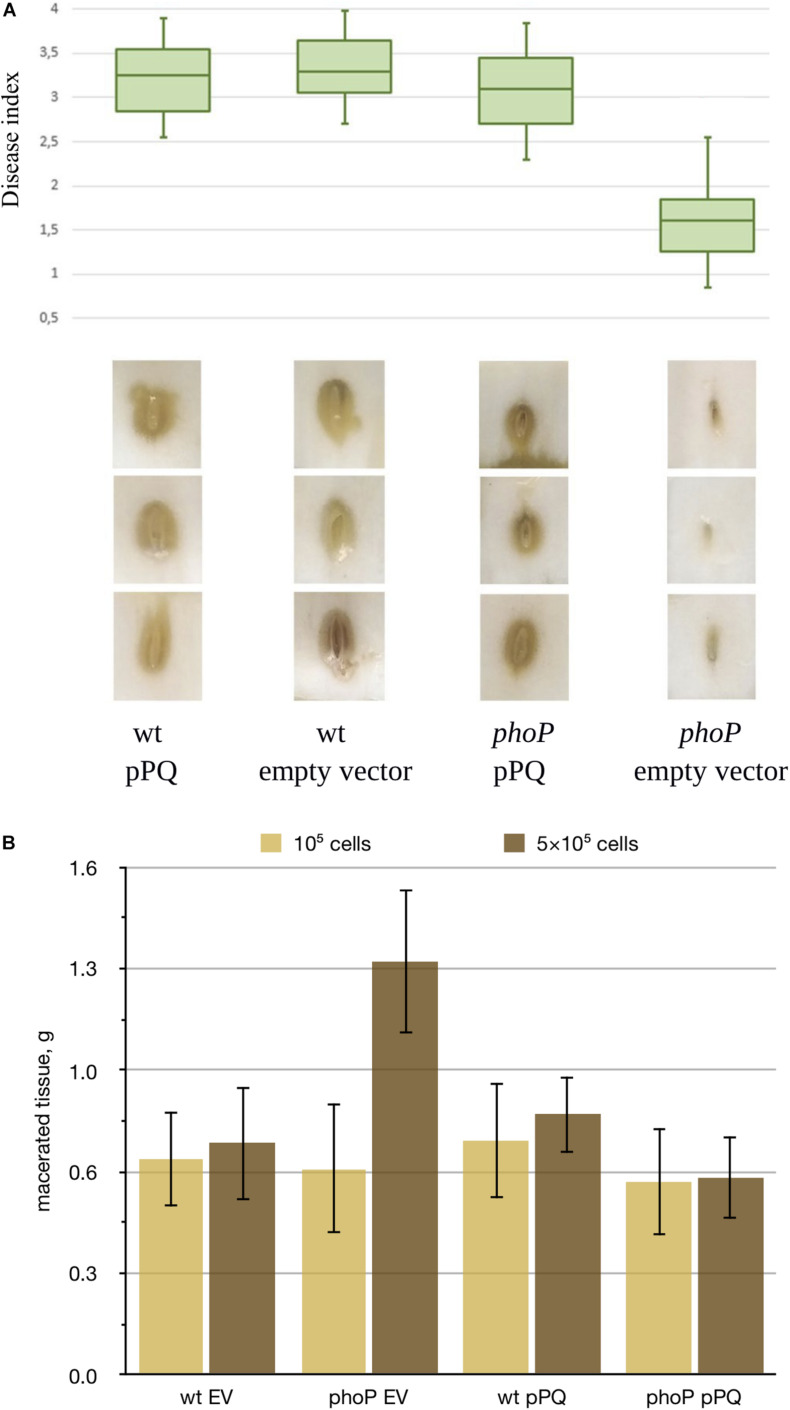
Effect of *phoP* inactivation on *Pve* virulence. **(A)** Boxplot representation of disease symptoms induced in Chinese cabbage leaves by the wild type *Pve* strain (wt) and its *phoP* mutant (phoP) carrying either an empty vector (EV) or the same vector with cloned *phoPQ* operon (pPQ). Middle bar = median; box limit = upper and lower quartile; extremes = minimal and maximal values. Disease index indicates: 0 no symptoms; 1-2 tissue damage mostly localised near inoculation site; 3-4 extensive tissue damage spreading sideways from the inoculation site. A Kruskal-Wallis *H*-test showed a statistically significant difference in disease indices between the strains (*p* = 0.002). Three typical leaf sections for each variant are shown below the box plot. **(B)** Potato tubers inoculated with JN42 wild type (wt) or *phoP* mutant bacteria carrying either the empty vector pZH449 (EV) or pZH449:*phoPQ* (pPQ). Two inoculum doses were used: 5⋅10^5^ or 1⋅10^5^ cells per inoculation site. 16 tubers were inoculated for each strain/inoculum dose combination. Macerated tissue was weighed at 48 h post-inoculation. Mean values with 95% confidence intervals are shown.

In Chinese cabbage virulence tests carried out at low inoculum doses (10^3^ cells), the mutant strain caused less damage than the wild type strain, and observed virulence defect was fully complemented by the plasmid expressing *phoPQ* (Figures 1A,B). We could not detect reproducible differences between the strains at higher inoculum doses (10^5^ cells) (data not shown). In potato tubers, we could reliably induce infection only starting with much higher inoculum dose (10^5^ cells) but no difference between the strains was observed. However, the *phoP* mutant generated 50% more macerated tuber tissue with a dose of 5 × 10^5^ cells (Figure 1B). In this case, the *phoP* mutant caused more tissue maceration than the wild type strain.

As the growth rates of the *phoP* mutant and the wild type strain in MMA were almost the same (Supplementary Figure 1), the differences in disease development show involvement of the PhoPQ two-component system in virulence regulation in *Pve*. Since low and high inoculum doses somewhat mimic the early and late stages of infection, virulence tests results could indicate a switch in the state of the PhoPQ two-component system during infection.

### PhoPQ System Controls Over a Hundred Genes in *P. versatile*

RNA-seq transcriptional profiling revealed 115 genes with expression level differing between the wild type and the *phoP* mutant strains. Most of the differentially expressed genes (DEGs) were activated, but 28 were repressed. Tables 1–4 and Supplementary Table 3 depict the genes, whose expression differed significantly (given a false discovery rate of 0.05) by at least twofold. The regulon composition and its control by PhoP are discussed in more detail in the following sections.

**TABLE 1:** PhoP-dependent genes of *Pve* involved in cell envelope modifications, tellurite resistance and anaerobic metabolism.

Gene name(s)	Locus_tag(s)	Fold change^a^	Product(s)	Best operator	Score^b^
**Lipid A remodelling**					
*visP*	OA04_00090	2.73	Virulence- and stress-related protein	TATTTTTTCTGTTGTTTCA	10.2
*–*	OA04_20060	3.18	Acyltransferase 3	CCGTTTAGCAACCGTTTAG	10.1
*lpxT*	OA04_26310	4.33	Lipid A 1-diphosphate synthase	TTTTTTCGTCTTTGTTTGG	8.4
*pgpB*	OA04_18890	5.59	Phosphatidylglycerophosphatase B	TTTTTTGAACATGGTTTTT	10.3
*ugd*	OA04_31420	49.63	UDP-glucose 6-dehydrogenase	ATGTTTAATTGGCGTTTAA	10.2
*arnBCADTEF*	OA04_31410-OA04_31350	78.53	4-amino-4-deoxy-L-arabinose transferase biosynthesis and lipid A modification	CCGTTTATCTGTAGTTTTA	8.95
**Tellurite resistance**					
*terW*	OA04_37880	6.92	Putative transcriptional regulator of terZABCDE operon	TGGTTTATCTCTCGTTGAA	9.8
*terZABCDE*	OA04_37940-OA04_37990	0.16	Tellurite resistance proteins	ATGTTTCATTAAGGTTTTT	8.8
*–*	OA04_10330	0.45	Inner membrane protein, TerC-like	TCGTTTACGTTTCGTTTAA	10.2
OA04_37930	OA04_37930	0.45	Hypothetical protein	ATGTTTCATTAAGGTTTTT	8.8
**Anaerobiosis-related genes**					
*focA*	OA04_25450	2.31	Formate transporter	AGTTTTATGGTTAATTTAT	7.9
*aegA_OA04_45440*	OA04_45430-OA04_45440	5.43	Oxidoreductase Fe-S binding subunit	TGTTTTTTGTCTGGTTTAC	10.8
*hybO*	OA04_12810	2.03	Hydrogenase 2 small subunit	TGATTTAACTATAATTTAA	10
*hypAB*	OA04_12910-OA04_12900	6.85	Hydrogenase nickel incorporation proteins HypA and HypB	TATTTTTCTACCTCTTTAT	8.6
*hydN*	OA04_13040	2.71	Electron transport protein HydN	TTGTTTTTTAATTATTTTA	11.7
*fdhF_2*	OA04_28980	2.36	Formate dehydrogenase H	TGTTTTTGACGATATTAAC	10.3
*nrdD*	OA04_04300	2.19	Anaerobic ribonucleoside triphosphate reductase	TATTTTGTCTTTTTTTTAG	8.8
*norV*	OA04_09050	2.55	Anaerobic nitric oxide reductase flavorubredoxin	CAGTTTTTAATTTGTTGAT	10.5
*fdnG*	OA04_14900	5.55	Formate dehydrogenase, nitrate-inducible, major subunit	ATGTTTTTTATTCGTTATA	8.1
*ccmA*	OA04_18220	5.88	Cytochrome c biogenesis protein CcmA	CAATATATTTCCGGTTTAA	7.7
*napF*	OA04_18370	3.94	Ferredoxin-type protein	TGTTTTTGTAGGGGTTACA	5.8
*glpA*	OA04_42210	3.14	sn-glycerol-3-phosphate dehydrogenase subunit A	TCGTTTAGTGTTCGTTTTT	8.6
*ubiU*	OA04_06900	5.99	Ubiquinone biosynthesis protein	TTTTTTATCAGTCGATGAA	7.3
*pepT*	OA04_23990	4.93	Tripeptidase T	CAGTTTTTTCCCGATTTAA	10.7
*grcA*	OA04_33700	6.06	Stress-induced alternative pyruvate formate-lyase subunit	GGTTTTAAAATTGATTTAA	10.5

*^*a*^Wild type vs. phoP mutant ratio. For operons, the value for the first gene is shown. Supplementary Table 3 contains values for all genes.*

*^*b*^ChIPmunk score for the best operator.*

**TABLE 2:** PhoP-dependent genes of *Pve* involved in pectin and arabinose degradation and utilisation.

Gene name(s)	Locus_tag(s)	Fold change^a^	Product(s)	Best operator	Score^b^
**Polygalacturonic acid utilisation**					
*pelI*	OA04_11360	0.14	Pectate lyase PelI	AATTTTATTATTTATTTAT	12.2
*pehA*	OA04_11370	117.60	Endo-polygalacturonase	AATTTTATTATTTATTTAT	12.2
*pehN*	OA04_12450	2.15	Endo-polygalacturonase	ATTTTTTAGTGAGGTTAAG	8.1
*pelP*	OA04_21040	9.67	Endo-pectate lyase	ATCTTTTCATTTTATTTAC	7.5
*pnl*	OA04_29050	2.63	Pectin lyase	TTATTTGTTTTTGATTAAA	9
*togT*	OA04_08060	2.01	Oligogalacturonide transporter	TATTTGATCTTGCGTTTAT	8.2
–	OA04_21060-OA04_21050	25.51	KdgM-like porins	ATTTTTTGTAATCATTTCG	8.9
–	OA04_07070	2.00	Polygalacturonic acid binding protein	–	–
–	OA04_12400	2.02	Coagulation factor 5/8 type domain protein	TTATTTTAAAGCGCTTTAC	7.8
–	OA04_32560	2.04	Coagulation factor 5/8 type domain protein	GTATTTTAAAGCGGTTTAC	8.7
**Arabinose transport and utilisation**					
*araD*	OA04_19040	0.08	L-ribulose-5-phosphate 4-epimerase	CTGTTTTTTTAGCGTTTCT	9.8
*araC*	OA04_22270	0.11	DNA-binding transcriptional regulator AraC	TATTTTATTTGCCATTTTG	9.1
*araFGH*	OA04_22300-OA04_22280	0.06	L-arabinose transporter subunits	GGTTTGTGCATACATTTAG	5.6
*araBA*	OA04_22310-OA04_22320	0.02	L-ribulokinase and L-arabinose isomerase	GGTTTGTGCATACATTTAG	5.6
*–*	OA04_38360-OA04_38370	0.19	Sugar (Glycoside-Pentoside-Hexuronide):cation symporter and exo-α-1,5-L-arabinofuranosidase	ATTTTTGCAACCGATTTCA	6.2
*ytfQRT*	OA04_43130-OA04_43110	0.14	Galactofuranose/arabinofuranose ABC transporter subunits	TGATTTTGTGCATATTTAC	7.3

*^*a*^Wild type vs. phoP mutant ratio. For operons, the value for the first gene is shown. Supplementary Table 3 contains values for all genes.*

*^*b*^ChIPmunk score for the best operator.*

**TABLE 3:** PhoP-dependent genes of *Pve* involved in transmembrane transport.

Gene name(s)	Locus_tag(s)	Fold change^a^	Product(s)	Best operator	Score^b^
**Iron import**					
*fetM*	OA04_28960	2.16	High affinity Fe^2+^ permease	GATTTTTACTGTTTTTTAG	8.2
*yiuABC*	OA04_33210-OA04_33230	8.36	Ferric-enterobactin ABC-transporter subunits	CTGTATACGCATTGTTTAT	9
–	OA04_26190	9.81	TonB-dependent receptor	TCGTTTAACTACGGTTTAT	12.7
*entC*	OA04_05590^c^	4.09	Enterobactin synthetase and transport proteins	TTTTTTTGTGCATGTTTCA	7.7
*fusB*	OA04_08790	2.29	TonB-like protein	CATTTGTATTATTATTTAT	10.7
–	OA04_13340	2.55	FecI-like RNA polymerase sigma factor	–	–
**Di- and tricarboxylate sensing and import**					
*citW*	OA04_25230	3.79	Citrate/acetate antiporter	GAGTTTTAAAGGCGTTTAT	8.1
*citM_OA04_29000*	OA04_28990-OA04_29000	0.07	CitMHS family citrate/H^+^ symporter and transcriptional regulator	TGGTTTAGACACCGTTTAA	10.2
–	OA04_29010	0.48	Sodium:dicarboxylate symporter	TAATTTTTTAATTATTTAA	10.6
*tcp*	OA04_13410	0.39	Methyl-accepting chemotaxis protein	TGTTTTTATTTGTGTTGAG	8.9
**Exporters**					
–	OA04_04830-OA04_04800	5.46	ABC-2 type exporter	CCGTTTAATTATCGTTTGC	10.5
–	OA04_05580	4.11	Putative efflux permease	TTTTTTTGTGCATGTTTCA	7.7
*alaE*	OA04_38680	2.79	L-alanine exporter	TGTTTTTAAAAAACTTTAA	7.6
–	OA04_40040	2.15	Putative efflux permease, MFS superfamily	CTATTTATCTCTTTTTATG	5.3
**Other transport**					
*mgtA*	OA04_05060	15.41	Magnesium transport ATPase	ACGTTGACGTCCGGTTTAG	6.2
–	OA04_25030	3.09	Glycine betaine/L-proline ABC transporter, ATPase subunit	–	–
–	OA04_22250	2.10	Putative inorganic ion transporter	TTTTTTACGCTCGATTTAC	9.8
*mtrB*	OA04_14870	2.16	Putative methylthioribose ABC transporter, periplasmic binding protein	CGTTTTATAAATTGATGTC	5.2
–	OA04_10630	2.31	ABC transporter substrate binding protein	CCGTTTTATTATTTTTTCG	8.6
*gcvB*	OA04_10700	2.17	GcvB RNA	–	–
–	OA04_29520	3.27	Extracellular solute-binding protein	GAGTATTACCACGATTTAC	6.3
–	OA04_29540^c^	3.39	ABC transporter permease	CGTTTTATTATTTATTTAT	13.1
–	OA04_42530	2.20	Extracellular solute-binding protein family 3	TTGTTTTAACGTGTTGTAA	6.4
–	OA04_45500	4.60	Extracellular solute-binding protein	TTATATTATAGCTATTTAT	8.6
–	OA04_05190	2.96	ABC transporter extracellular solute binding protein	–	–
–	OA04_02820	2.89	Amino acid ABC transporter substrate binding periplasmic protein	ACGTTGTGTGATGATTTAT	7.8

*^*a*^Wild type vs. phoP mutant ratio. For operons, the value for the first gene is shown. Supplementary Table 3 contains values for all genes.*

*^*b*^ChIPmunk score for the best operator.*

*^*c*^Only the first gene(s) of the larger operon shows differential expression.*

**TABLE 4:** PhoP-dependent genes of *Pve –* regulators and unclassified.

Gene name(s)	Locus_tag(s)	Fold change^a^	Product(s)	Best operator	Score^b^
**Regulators**					
*glnK*	OA04_12080	0.49	Nitrogen regulatory protein P-II	–	–
*-*	OA04_01500	0.34	LysR family transcriptional regulator	–	–
*sftR*	OA04_43750	2.34	LysR family transcriptional regulator SftR	CGGTTTTATTATTTTTTCT	9.4
*cbl*	OA04_28870	2.71	Transcriptional regulator CysB-like protein	CCATTTTGCTATGATTTAT	10.8
**Miscellaneous**					
*scrK*	OA04_04060	0.45	Fructokinase	–	–
*yjbJ*	OA04_05950	2.0	Osmotic stress-induced protein| RpoS regulon	–	–
*ygdBppdD*	OA04_10430-OA04_10440	3.58	Putative pilins	–	–
*ftp*	OA04_17930	2.29	Periplasmic FAD:protein FMN transferase	TTTTTTTCCTTTCATTTGT	10.3
*slyB*	OA04_18750	2.46	Outer membrane lipoprotein	CTGTTTATACGCAATTTAA	9.9
*ychH*	OA04_21530	2.31	Putative inner membrane stress-induced protein	AATATTTTTTTACGTTTAA	6
*ynfK*	OA04_22140	5.06	Putative dithiobiotin synthetase	CATTTTAGCTGTGCTTGTT	5.7
–	OA04_23860	3.76	hypothetical protein	GGGTATTAATCTTGTTTAA	7.3
–	OA04_24930	2.36	Protein kinase-like domain protein	ATATTTGATCTGCGTTGAA	5.4
–	OA04_28020	2.03	2-dehydropantoate 2-reductase	–	–
–	OA04_29260	67.65	Alpha/beta hydrolase superfamily protein	CTATTGAGCCGTGGTTTAA	8.4
–	OA04_29530	2.22	Putative 2-methylcitrate dehydratase	CGTTTTATTATTTATTTAT	13.1
–	OA04_29750	2.91	Putative acireductone dioxygenase	TTTTGTTATTTCGATTTAA	8
–	OA04_32550	3.02	Exported choloylglycine hydrolase	TTGTTTATTTTAGGTTTAT	13.8
*raiA*	OA04_34460	2.18	Stationary phase translation inhibitor and ribosome stability factor	CCGTTTTTTTTATGGTTAG	7.3
*gudX*	OA04_36650	4.2	Glucarate dehydratase	ACTTTTTACTGAGGTTGGT	7.5
*metF*	OA04_43310	3.58	5,10-methylenetetrahydrofolate reductase	–	–

*^*a*^Wild type vs. phoP mutant ratio. For operons, the value for the first gene is shown. Supplementary Table 3 contains values for all genes.*

*^*b*^ChIPmunk score for the best operator.*

The RNA-seq data also confirmed the absence of *phoP* expression in the mutant cells. Expression of *phoQ* located immediately downstream in the same operon was barely detectable in the *phoP* mutant, which is probably due to the polar effect of *phoP* inactivation. Expression levels of the selected genes from different functional categories were verified by qPCR and found to correlate well with RNA-seq data (Supplementary Figure 2).

**FIGURE 2:**
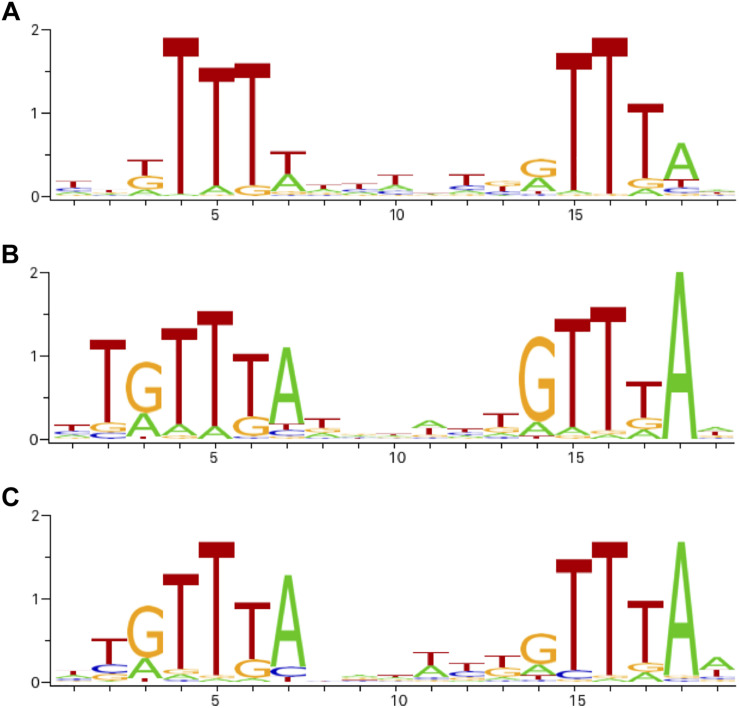
Sequence logos of operator motifs for three PhoP orthologues. **(A)** *P. versatile* motif made from the sites listed in Table 1. **(B)** *E. coli* motif (combined non-redundant RegulonDB and CollecTF data). **(C)** *S. enterica* motif (CollecTF data).

We looked for the presence of PhoP binding sites in the regulatory regions of the differentially expressed genes. ChIPMunk (Kulakovskiy et al., 2010) could locate a relatively weakly conserved 19 bp sequence containing two direct gTTTa repeats (Figure 2A) within most of the 85 regulatory regions of the differentially expressed genes. This sequence resembles the PhoP binding sites reported for other enterobacteria (Figures 2B,C) and was therefore considered the likely PhoP binding site.

### The *P. versatile* PhoP Regulon Part Conserved in *Enterobacterales*

The universally conserved core of enterobacterial PhoP regulons is known to include only three genes: *phoP*, *phoQ*, and *slyB* (Perez et al., 2009). In *Pve*, there are high-scoring PhoP-binding sites in front of both the *phoPQ* operon and the *slyB* gene. The expression level of *slyB* is decreased 2.5 times in the *phoP* mutant (Table 1). Therefore, these three genes are highly likely to be directly controlled by PhoP in *Pve.*

Additional PhoP regulon members shared by *P. versatile* with some other enterobacteria are required for cell envelope modifications that improve resistance to antimicrobial peptides and oxidative stress. Most of these modifications alter the structure of the lipid A domain of the outer membrane lipopolysaccharide (LPS). These alterations are known to affect pathogenesis by changing outer-membrane permeability, increasing resistance to antimicrobial peptides and interfering with the ability of the host to recognise LPS as a conserved microbe-associated molecular pattern (see Needham and Trent, 2013, for a review).

The strongest impact (up to 78x decrease of expression levels) *phoP* inactivation had on the eight genes responsible for the attaching 4-aminoarabinose to the lipid A domain of the LPS. These genes are organised in *Pve* into two tightly linked operons: monocistronic *udg* and the *arnBCADTEF* operon located just after *ugd*. Multiple high scoring PhoP binding sites are located in front of both *ugd* and *arnB*, ensuring direct PhoP control over the whole locus. The addition of 4-aminoarabinose to lipid A reduces the negative charge of the outer membrane and makes the cell more resistant to the CAMPs (Needham and Trent, 2013).

Expression of four more LPS-related genes is activated by PhoP 3–6 fold. The product of OA04_20060 ORF belongs to the acyltransferase 3 family (PF01757) suggesting it might be involved in O-acetylating peptidoglycan (PG) thus increasing resistance to lysozyme (Slauch et al., 1996; Bera et al., 2004). *visP* (*ygiW*) homologue from *S. typhimurium* codes for a virulence- and stress-related protein which binds to peptidoglycan and is required for polymyxin B resistance (Moreira et al., 2013). *lpxT* codes for the lipid A 1-diphosphate synthase (Touzé et al., 2008b) which increases lipid A negative charge and therefore counteracts the action of *arn* gene products. *pgpB*has undecaprenyl-pyrophosphate phosphatase activity, required for the biosynthesis of the lipid carrier undecaprenyl phosphate (Touzé et al., 2008a). Since LpxT uses undecaprenyl pyrophosphate as a phosphate donor to phosphorylate lipid A (Touzé et al., 2008b), PhoP-dependent PgpB activation might reduce phosphate donor availability for LpxT and hence prevent (or reduce) increase in negative charge of lipid A.

**FIGURE 3:**
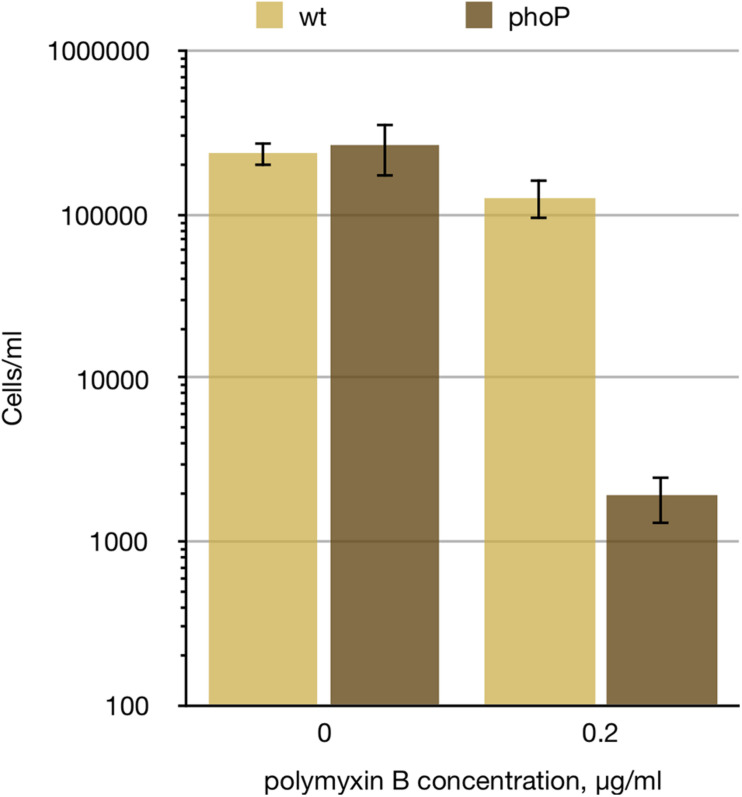
*phoP* is required for *Pve* polymyxin resistance. Wild type and *phoP* mutant *Pve* cultures were grown aerobically in LB medium to mid-log phase, diluted to 10^5^ cells ml^–1^ and split in two. 0.2 μg/ml of polymyxin B was added to one of the split cultures and incubation continued for 4 more hours. Surviving cell numbers were determined by plating. Mean values of three biological replicates and 95% confidence intervals are shown.

As the products of the *ugd-arn* locus, VisP, and LpxT have the opposite effects on the lipid A negative charge and the CAMP resistance (and PgpB activity adds to the situation complexity), we have checked the resistance of *P. versatile* to cationic peptide polymyxin B and found it to be drastically reduced in the *phoP* mutant (Figure 3). Polymyxin resistance was restored in the *phoP* mutant to the wild type levels by a plasmid expressing the full phoPQ operon (data not shown). Thus, the net phenotypic result of the PhoP-activated remodelling of the cell envelope in *Pve* is the activation of the CAMP resistance.

### Pectin Degradation and Utilisation

Degrading pectic compounds of plant cell walls is a characteristic feature of pectinolytic bacteria that is directly responsible for maceration of host plant tissues. PhoP was found to regulate a selection of pectinolysis related genes, activating 11 and repressing one of them.

Just as in *P. parmentieri* (Flego et al., 2000), PhoP is required in *Pve* for efficient transcription of the *pehA* gene encoding the major endo-polygalacturonase. *pehA* expression demonstrates the strongest response to *phoP* inactivation among all DEGs (118x), which correlates with the presence of multiple PhoP binding sites in the *pehA* regulatory region.

Three more PhoP-activated genes code for pectinolytic enzymes: periplasmic endo-pectate lyase PelP (9.7x activation),endo-polygalacturonosidase PehN (2.2x activation) and pectin lyase Pnl (2.6x activation).

Three oligogalacturonate transporter genes are also under positive PhoP control: the oligogalacturonate/cation symporter *togT* (2x activation) and two neighbour loci OA04_21050 and OA04_21060 (9.1x and 25.5x activation) coding for porins with weak homology to oligogalacturonate-specific porins KdgM and KdgN. The major oligogalacturonate transport and utilisation locus *kdgF-kduDpelWtogMNAB-kdgM* also has PhoP binding sites in the appropriate regulatory regions, but the observed expression level differences were slightly below the threshold (data not shown), suggesting possible input of additional regulator(s) into transcriptional control of this locus.

Three uncharacterised genes with about twofold activation by PhoP (*OA04_07070*, *OA04_12400*, and *OA04_32560*) encode proteins with family 32 carbohydrate-binding module (CBM32). One well-characterised member of this family, YeCBM32 from *Yersinia enterocolitica*, binds to polygalacturonic acid (Abbott et al., 2007) and was suggested to bind and retain polygalacturonic acid (PGA) within the periplasm as a substrate for further depolymerisation (Abbott and Boraston, 2008). YeCBM32 has sufficient homology (over 72% identity) to OA04_07070 to assume orthology between the two proteins. Additional evidence for the involvement of *OA04_07070* in pectin utilisation is provided by the presence of the signal peptide in the product and a binding site for the major pectinolysis regulator KdgR in front of this gene. Two more proteins with CBM32 are larger, have no signal peptide and their amino acid identity to YeCBM32 is very weak suggesting different function.

Overall, PhoP appears to be a strong activator of extracellular and outer membrane components and a weak activator of periplasmic and cytoplasmic membrane components of polygalacturonate utilisation system. Together, PhoP-activated genes code for a complete set of proteins required for polygalacturonate depolymerisation and transport into the bacterial cytoplasm. However, *Pve* has more genes encoding PGA depolymerases that showed no PhoP dependence in our experiment. In particular, expression levels of the genes in the major pectate lyase gene cluster (*pelA, pelB, pelC, pelZ*) were the same in the wild type strain and the *phoP* mutant. Therefore, we speculate that PhoP activates a subsystem of PGA depolymerisation and transport, finely tuned for a specific condition or a specific variant of PGA modification.

*pelI* is the only pectinolysis gene repressed (7x) by PhoP. It is located next to *pehA*, is transcribed in the opposite direction and shares its regulatory region with *pehA*. PelI is known to be strongly expressed *in planta* (Jafra et al., 1999). It was also reported to induce a hypersensitive response in plants (Shevchik et al., 1998). Since strong *pelI* expression can be expected to occur only when the PhoPQ system is inactivated, high *pelI* expression level *in planta* may indicate such an inactivation.

### Citrate and Dicarboxylate Transport

Two genes of citrate transporters are controlled by PhoP: *citM* (14x repressed) and *citW* (3.8x induced).

*citW*, coding for citrate/acetate antiporter, is located within the citrate fermentation locus. The locus includes *citAB* operon encoding a citrate responsive two-component system and a divergently transcribed *citW*, which is followed by the *citYCDEFXG* operon (coding for the subunits of citrate lyase). All these genes (including *citW*) were poorly expressed in our study. *citW* and the whole citrate fermentation locus is expected to be relevant for citrate utilisation under anaerobic conditions while *citM*, coding for a citrate/proton symporter operates in aerobic environment (Bott, 1997; Kästner et al., 2002).

Besides the transporters and regulators, the *tcp* gene encoding citrate chemotaxis receptor is activated threefold in the *phoP* mutant.

One more transporter gene, *OA04_29010*, is located downstream of the *citM* operon and codes for a putative sodium:dicarboxylate symporter. Just as the preceding operon, *OA04_29010* is negatively controlled by PhoP (2.1x repression).

Citrate and dicarboxylates (e.g., malate) are abundant in plant cells, can be excreted into either apoplast or soil (Meyer et al., 2010) and therefore constitute good nutrients for a plant pathogen. In *P. atrosepticum*, citrate uptake was attributed to a highly specific transporter Cit1 and shown to be important for virulence (Urbany and Neuhaus, 2008). Cit1 orthologue was expressed poorly underconditions used in this work, so we could not evaluate the involvement of PhoP in its control. However, a good PhoP binding site was located in front of *cit1*. High scoring PhoP binding sites were also located in the regulatory regions of almost all dicarboxylate transporter genes annotated in the *Pve* genome: *dctPQM*, *dcuA*, *dcuC*, *dcuB*, and *dctA* (more detail available in the “Extended regulon” section). We expect PhoP to affect the expression of at least some of these dicarboxylate transporters in different conditions, e.g., in the presence of dicarboxylates.

Overall, PhoP can be considered to be an important regulator of di- and tricarboxylate transporters. As some of these transporter genes are under negative control while others—under positive control, we speculate that PhoP may be involved in switching between the forms of carboxylate transporters appropriate for different environmental conditions including different stages of infection.

### Arabinose Transport and Utilisation

Thirteen genes related to transmembrane arabinose transport and utilisation are organised into five or six operons in *Pve* and are strongly repressed by PhoP (Table 1). The main arabinose uptake and catabolism locus in *Pve* includes the divergently transcribed *araFGH* (transporter) and *araBA* (catabolism) operons. The *araC* gene is separated from *araH* by a 203 bp gap permitting an independent transcription initiation, but the lack of an obvious transcription terminator in this gap can also allow co-transcription with the upstream *araFGH* genes. The unlinked monocistronic *araD* codes for the last of the three enzymes required for arabinose catabolism.

The *ytfQRTyjfF* operon encodes a periplasmic sugar-binding protein YtfQ and three subunits of an ABC transporter. YtfQ was shown to specifically bind the furanose forms of arabinose and galactose (Horler et al., 2009). The *OA04_38360*-*OA04_38370* operon codes for the putative glycoside-pentoside-hexuronide:cation symporter and glycosyl hydrolase which belongs to the subfamily 43.26 according to CAZy classification (Lombard et al., 2014). Well characterised bacterial members of this subfamily with sufficient homology to *OA04_38360* product were described as exo-α-1,5-L-arabinofuranosidases specialised at cleaving short arabinooligosaccharides (Matsuo et al., 2000, p. 901; Fujimoto et al., 2010, p. 43; Linares-Pastén et al., 2017). Arabinose is present in plant cell walls mainly in the form of arabinofuranosyl residues of the cell wall polysaccharides and proteoglycans such as pectic arabinan, arabinoxylan, and arabinogalactan-proteins (Kotake et al., 2016). Presumably, the *OA04_38360*-*OA04_38370* and *ytfQRTyjfF* operon products are involved in transport and utilisation of arabinofuranose containing products of plant cell wall breakdown.

As the *OA04_38370* product does not have a signal peptide, it can only degrade cytoplasmic substrates such as α-L-Ara*f* containing oligo- or disaccharides which could be released from plant cell wall by the action of other enzymes. At least two *Pve* enzymes are relevant: a putative arabinogalactanase encoded by *OA04_08560* and an arabinogalactan endo-1,4-beta-galactosidase encoded by *ganA* of the *ganEFGAB* operon. Although no differential expression was registered *in vitro* for any of the two loci encoding secreted arabinogalactanases, both arabinogalactanase genes could be expected to be induced by galactose *in planta.* Indeed, our recent transcriptome profiling of *P. atrosepticum* has shown 4- and 24-fold *in planta* induction of *OA04_08560* and *ganA* orthologues *ECA0852* and *ECA3128* (Gorshkov et al., 2018).

High scoring PhoP binding sites are located near two out of six arabinose related differentially expressed transcriptional units. A PhoP binding site overlaps the very beginning of the *araD* reading frame, suggesting direct negative control of *araD* by PhoP. Another binding site was found in front of the *araC* gene in a position suitable for repression. Three rather weak PhoP binding sites were found in different ChIPmunk runs within the regulatory region of the *OA04_38360*-*OA04_38370* operon, suggesting the likelihood of direct PhoP control over this operon as well.

**FIGURE 4:**
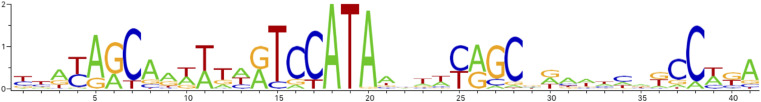
AraC binding site sequence logo.

Since *araC* codes for the arabinose-responsive transcription factor, we searched for AraC binding sites in the *Pve* genome. First, the AraC operator profile was constructed. For this, we downloaded the AraC_Enterobacterales motif from RegPrecise (Novichkov et al., 2013). However, this short (17 bp) motif corresponds to just a part of the binding site. AraC has two DNA binding domains, both of them can contact DNA and the active AraC form is dimeric (Schleif, 2010), so the real binding site should be larger. We checked the conservation around the sites annotated in RegPrecise and found the conserved region to cover about 40 bp—sufficient to allow binding of four DNA binding domains of the dimeric AraC. The extended part of the motif is less conserved but has the same orientation and an overall resemblance to the conserved part (Figure 4 and Supplementary File 3). This configuration of the operator is suitable for the binding of a tandem of AraC subunits in their activating conformation. The most conserved part of this motif corresponds well to the one recently defined experimentally (Stringer et al., 2014).

Scanning of the *Pve* genome with the AraC operator profile has found matches in three locations: in the regulatory region between the *araBA* and *araFGHC* operons (two sites), in front of the *araD* gene and the *ytfQRTyjfF* operon. Therefore, PhoP dependence of differentially expressed arabinose-related transcriptional units could be explained by direct negative control by PhoP over *araD*, *araC, ytfQRTyjfF* and *OA04_38360*-*OA04_38370*, combined with positive control via AraC over *ytfQRTyjfF, araD* and the other two operons that lack PhoP sites in their regulatory regions (*araBA*, *araFGH*).

**FIGURE 5:**
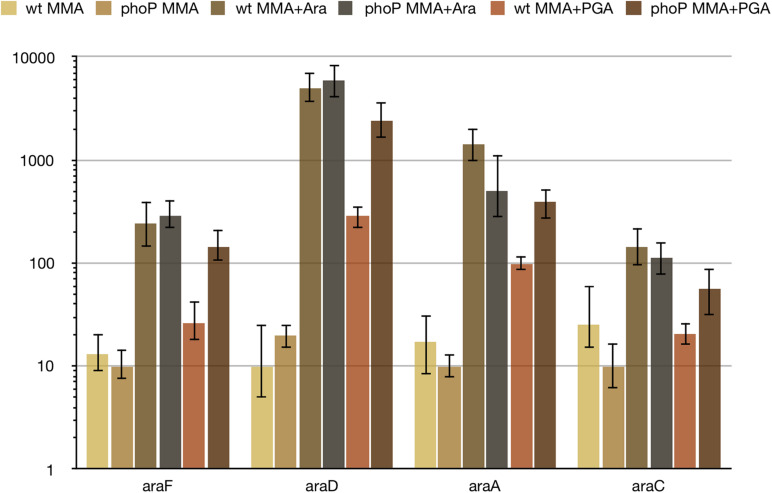
Differential expression of *ara* genes depends on the presence of polygalacturonate. For qPCR measurement, RNA was isolated from the wild type (wt) or *phoP* mutant *Pve* cells grown in MMA, MMA with 0.5% arabinose (MMA + ara) or in MMA with 0.5% sodium polypectate (MMA + PGA). Mean values of relative expression levels with 95% confidence intervals are shown.

Further investigation of PhoP-dependent control over arabinose utilisation genes has shown, that (i) arabinose is a strong inducer of *ara* genes and (ii) the observed differential expression depends on the presence of sodium polypectate in the medium (Figure 5). Without polypectate, expression of *ara* genes does not require PhoP, both with and without arabinose. Sodium polypectate appears to be a weak inducer of arabinose-related operons in the wild type strain, but induction is much stronger in the *phoP* mutant (Figure 5). PhoPQ two-component system does not seem to respond to polypectate, as most of the other DEGs have the same expression with and without polypectate (data not shown). This suggests indirect regulation by another PhoP-controlled and polypectate responsive transcriptional factor and requires further investigation.

### Other Transporters

PhoP is known to be involved in the regulation of at least one of the Mg^2+^ transporters in enteric bacteria (Perez et al., 2009). *P. versatile* seems to have the only dedicated Mg^2+^ transporter encoded by *mgtA*. In the conditions of our RNA-seq experiment with moderate (0.5 mM) Mg^2+^ concentration *mgtA* was weakly expressed in the wild type cells and was almost silent in the *phoP* mutant. At least 10-fold higher expression of the 5′ UTS compared to the coding frame of *mgtA* (Supplementary Figure 3) hints to a riboswitch-like control of transcription elongation into the coding region of *mgtA* analogously to the mechanism described for *S. enterica* (Cromie and Groisman, 2010).

PhoP activates several genes and operons involved in iron acquisition. Since the RNA-seq experiment was performed without iron limitation, these genes had a rather low expression and differential expression in many cases was detected only for the first gene of multigene operons. DEGs related to iron scavenging include *fetM* encoding high-affinity Fe^2+^ permease, *yiuABC* coding for ferric-enterobactin ABC transporter and OA04_26190 encoding TonB-dependent outer membrane siderophore receptor. *entC* is the first gene of the enterobactin-like siderophore synthesis operon *entCEBFA*. *fusB*, the first gene of the *fusBACD* operon, encodes a TonB-like protein known to be required for import of plant ferredoxin (Wojnowska and Walker, 2019). Differentially expressed FecI-like sigma-factor gene *OA04_13340* resides within an apparent TonB-dependent iron acquisition locus of eight genes and is likely to control its transcription.

Three PhoP regulated transporters participate in amino acid import. The *OA04_29540-OA04_29570* operon is likely involved in the import and modification of branched-chain amino acids. *OA04_42530* codes for the periplasmic binding protein of one more amino acid transporter. OA04_02820 product is annotated as a cystine-binding periplasmic protein.

Three more PhoP-activated loci encode putative efflux transporters. These include L-alanine exporter AlaE (OA04_ 38680), putative multidrug efflux transporter OA04_04830-OA04_04800 and AraJ family efflux permease OA04_40040.

Three additional transporters do not belong to any of the groups mentioned above. *mtrB* codes for the periplasmic binding subunit of methylthioribose ABC transporter. *OA04_25030* encodes the ATPase subunit of glycine betaine/L-proline ABC transporter, *OA04_22250*—the putative sodium/bile acid cotransporter. The last transporter may act in conjunction with choloylglycine hydrolase encoded by *OA04_32550*, which is also activated by PhoP.

The potential substrates of four other transporters (OA04_10630, OA04_29520, OA04_45500, and OA04_05190- OA04_05160) could not be inferred even approximately.

All the genes coding for the transporter subunits described above are activated by PhoP. However, the observed effect of *phoP* inactivation may be indirect in many cases. In particular, we found no PhoP binding sites near *mtrB*, *OA04_25030, OA04_05190* and only weak sites could be located for *OA04_38680*, *OA04_45500*, and *OA04_02820*.

We also note that the GcvB ncRNA, a well-known negative regulator of amino acid transporter genes (Sharma et al., 2011), is 2.2x activated. This effect may partially compensate for the decrease of the transporter gene expression in the *phoP* mutant. We could not find a PhoP binding site in front of *gcvB.* Therefore, *gcvB* expression control by PhoP might be indirect, just as has been reported for *E. coli* (Raghavan et al., 2011). GcvB has also been reported to control PhoP expression posttranscriptionally by both destabilising its mRNA and decreasing its translation (Coornaert et al., 2013). Such reciprocal repression between PhoP and GcvB, if present in *Pve*, might result in amplification of any changes in expression of either regulator.

### Tellurite Resistance Genes

A group of seven genes (*terZABCDE* and divergently transcribed *OA04_37930*) tentatively annotated as coding for tellurite resistance proteins is 2-6x repressed by PhoP. *OA04_37930* is the first gene in the operon of five uncharacterised genes (1.7-1.9x repression of the four downstream genes). These genes are a part of a large locus of 18 presumably functionally linked genes (Anantharaman et al., 2012) organised into four or five operons (Supplementary Figure 4). Although the exact function of this locus *in vivo* is not clear, orthologous genes are required for tellurite resistance in some bacteria (Whelan et al., 1995, 1997; Valková et al., 2007). Tellurite is also thought to induce oxidative stress and tellurite resistance might be the result of decreased sensitivity to the oxidative stress (Chasteen et al., 2009).

At least one PhoP binding site is located in the regulatory region between *terZ* and *OA04_37930*, one more is present upstream of the *terW* gene. *terW* (6.9x induced) is located a few genes away and was shown to be responsible for transcriptional control of *ter* genes in *E. coli* (Valkovicova et al., 2011). The part of the *ter* operon regulatory region most conserved between *E. coli* and *Pectobacterium* sp. sequences contains a 25 bp palindrome (Supplementary Figure 4 and Supplementary File 3). Its position corresponds to the region protected by TerW (Valkovicova et al., 2011) and probably corresponds to the TerW binding site. Three putative TerW binding sites could be located within the *ter* cluster in *Pve*: two in the middle of the intergenic region between the divergently transcribed operons *(terZABCDE* and *OA04_37930-OA04_37890*) and the third one is in front of *terY1*. Thus, either the direct repression or a transcriptional cascade, where PhoP activates *terW* and TerW represses divergent operons, are responsible for the observed PhoP dependence of the genes in the *ter* locus. Since the *terY* operon does not respond to *phoP* inactivation, direct transcriptional control of PhoP over other *ter* genes is more likely in the conditions studied.

The *OA04_10330* gene (2.2x repression) is unlinked to the main *ter* locus, but codes for a TerC-like membrane protein and may be functionally linked to the *ter* genes.

*Pve* cells growing on the medium with low potassium tellurite concentration form characteristic black colonies, indicating the reduction of tellurite to tellurium. We did not notice a significant difference in black colouring between the wild type *Pve* and the *phoP* mutant, but the *phoP* mutant could grow in the MMN medium with potassium tellurite concentration 3 μg ml^–1^ that is inhibitory to the wild type strain (data not shown).

The actual role of “tellurite resistance” genes of a soft rot pathogen requires separate investigation. Soft rot bacteria can hardly ever encounter significant concentrations of tellurite in their natural environment, so these genes are likely responsible for coping with some other stress. Searching the nr and wgs subdivisions of GenBank shows that “tellurite resistance” genes are present in only three pectobacterial species: *P. versatile*, *P. parmentieri*, and *P. polaris* suggesting such stress is important for the lifestyles of these species.

### Anaerobiosis-Related Genes

The transcription profiling experiment described here was performed in aerobic conditions, and most of the anaerobiosis-related genes were expressed poorly. However, the PhoP-dependent positive control over important aspects of anaerobic metabolism was noticeable. The largest group of PhoP-activated genes is required for the detoxification of formate produced by enteric bacteria in anaerobic fermentative conditions. These genes include the formate transporter gene *focA* and several subunits of formate hydrogenlyase. Expression of the *aegA* operon, coding for formate metabolism proteins, is also PhoP-dependent. *aegA* codes for an oxidoreductase that was recently shown to be involved in formate-dependent catabolism of urate (Iwadate and Kato, 2019). A gene downstream *aegA*, *OA04_45440*, codes for an uncharacterised [4Fe-4S] ferredoxin subunit of hydrogenase or formate dehydrogenase.

Weakly expressed operons within the hydrogen metabolism gene cluster were PhoP dependent. These include three operons. The first operon is *hybOABCDE*, coding for subunits of hydrogenase 2. The second one is a *hypABCDE* operon coding for hydrogenase maturation proteins. The third operon includes genes coding for electron transport protein, which is required for formate dehydrogenase activity (*hydN*), formate dehydrogenase H (*fdhF*) and one more hydrogenase maturase (*hypF*). The *Pve* 3-2 genome carries a second *fdhF* paralogue (*fdhF_2*) which is also PhoP activated. The *fdnGHI* operon, coding for nitrate inducible formate dehydrogenase N, is PhoP activated too.

Anaerobic nitrate reduction may also be controlled by PhoP. The targets include a PhoP-activated *nap* operon responsible for periplasmic nitrate reductase production (differential expression was registered for only the first gene *napF*). Clear differential expression was detected for *ccmA*, the first gene of the *ccmABCD* operon coding for the subunits of the heme trafficking system. This system delivers heme to holocytochrome *c* synthase CcmFGH. And since this cytochrome *c* biogenesis system is required for maturation of cytochrome *c* subunits of nitrate and nitrite reductases, PhoP might be required for anaerobic nitrate and nitrite reduction. An additional level of PhoP control over nitrite reduction is provided via regulation of formate dehydrogenase that serves as the electron donor for formate-dependent nitrite reductase coded for by the *nrf* operon (poorly expressed in this experiment). PhoP also appears to control the *norVnorW* operon coding for anaerobic nitric oxide reductase.

Anaerobic respiration with fumarate as an electron acceptor may also be PhoP dependent due to control of at least one key gene, *glpA*. The *glpABC* operon codes for subunits of anaerobic glycerol-3-phosphate dehydrogenase, which is required for anaerobic respiration with fumarate as a terminal electron acceptor.

Three more PhoP-controlled genes contribute to important aspects of anaerobic metabolism. The *nrdD* gene codes for anaerobic ribonucleoside triphosphate reductase, which is essential in *E. coli* for deoxyribonucleotide synthesis during strict anaerobic growth (Garriga et al., 1996). *ubiQ* is required for anaerobic ubiquinone biosynthesis (Pelosi et al., 2019). GrcA restores pyruvate formate-lyase (PFL) activity after oxidative damage to the main PFL subunit (Wagner et al., 2001; Bowman et al., 2019) and thus may help the pathogen to maintain the high activity of key glycolysis enzyme at microaerobic conditions.

### Regulators

Nine regulatory genes showed differential expression in our experiments. Three of the transcription factors (AraC, TerW and OA04_13340) and the regulatory RNA GcvB were already discussed above. *glnK* codes for the second nitrogen regulatory protein P-II. Since it is functionally equivalent to P-II encoded by *glnB*, and *glnB* expression level is much higher (data not shown), the significance of the twofold difference of *glnK* expression levels should be minor.

Three regulators belong to the LysR family of transcription factors: OA04_01500, SftR and Cbl. OA04_01500 is a member of the RpoN regulon, had very low expression in the nitrogen-rich conditions of this experiment and was therefore unlikely to contribute significantly to the observed expression values.

A LysR family TF annotated as SftR is 2.3x activated by PhoP. We located the only potential operator in *Pve* genome that might be bound by SftR in the *atsR*-*atsBCA* intergenic region (data not shown). Therefore, SftR involvement in the control of the DEGs identified in this experiment is unlikely.

*cbl* (2.7x activation) codes for a CysB-like protein, whose closest homologue in *E. coli* controls genes required for aliphatic sulphonate and taurine utilisation and homeostatic response to sulphate starvation, according to RegulonDB. However, the ligand-binding domains of the homologues show low similarity, unlike the highly similar DBDs, which suggests a possibility of binding different ligand(s) by these two TFs. CysB-like proteins were reported to control functions unrelated to sulphur utilisation (Delic-Attree et al., 1997; Imperi et al., 2010; Farrow et al., 2015). A rather high transcription level of *cbl* in *Pve* hints at a possibility that this TF controls some DEGs found in this experiment.

One more well-expressed transcription factor belongs to the GntR family. It is coded for by the *OA04_29000* gene located downstream of *citM*, probably in the same operon. The involvement of *OA04_29000* and *cbl* gene products in transcriptional control of the DEGs described here is currently under investigation and will be reported separately.

### The Extended PhoP Regulon

Since PhoP is a global transcriptional regulator, PhoP-dependent genes differentially expressed in the single condition studied in this work probably constitute just a subset of the PhoP regulon. To see a broader picture, we analysed potential PhoP operators located near transcriptional units that did not pass differential expression thresholds. Since operator motif based on our experimental dataset (Table 1 and Figure 2A), had relatively low information content, we have looked for the possibility of applying search profiles based on *S. enterica* and *E. coli* data which cover a more diverse set of conditions.

**FIGURE 6:**
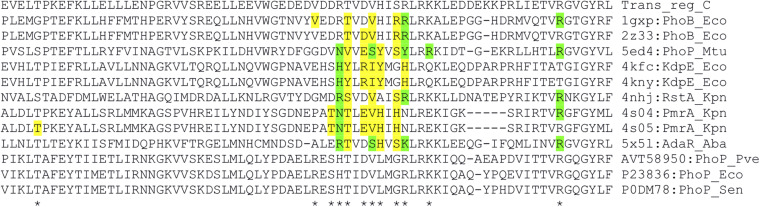
Alignment of DNA binding domain sequences of the OmpR family TFs. The sequences are aligned with hmmalign according to the Trans_reg_C (PF00486) family model. The amino acid residues of OmpR family proteins that form direct contacts with nucleotide bases were determined by the interaction service of NPIDB (Zanegina et al., 2016). The residues making specific contacts with DNA bases are highlighted in green (hydrogen bonds) and yellow (hydrophobic contacts). Positions with contact observed in at least one sequence are marked with an asterisk. PDB/GenPept/Uniprot IDs followed after a colon by protein names combined with species abbreviations are shown to the right of each sequence. Species names are abbreviated as Eco (*Escherichia coli*), Mtu (*Mycobacterium tuberculosis*), Kpn (*Klebsiella pneumoniae*), Aba (*Acinetobacter baumannii*), Pve (*Pectobacterium versatile*), and Sen (*Salmonella enterica*).

First, we have analysed the structures of TF-operator complexes for OmpR family proteins that are present in Protein Data Bank (Berman, 2000). This analysis allowed us to locate the positions of amino acid residues responsible for making specific contacts with DNA bases (Figure 6). Aligning to homologues with known structures shows that PhoP proteins from *Pve, E. coli*, and *S. enterica* have identical amino acid residues in the positions making specific contacts with DNA bases (Figure 6). This strongly suggests that indicated proteins must recognise very similar or even identical operator sequence specificities. The differences between their reported operator motifs may reflect the differences between the data sets and algorithms used for their inference. Therefore, the search for additional PhoP binding sites was done by scanning *Pve* genome sequence with four PhoP operator profiles (Supplementary File 1): the ones based on our experimental data, *E. coli* data, *S. enterica* data and profile inferred *in silico* as described in “Materials and Methods” section.

The scan identified a total of 812 genes organised into 254 transcriptional units that could be the targets of transcriptional control by PhoP (Supplementary File 2).

Many of these putative PhoP regulon members are related to plant cell wall degradation and utilisation of carbon sources abundant in plants. Of note are the genes/operons encoding pectate lyases (*pelA* and *pelB*), pectin methyl/acetyl esterases (*pmeB* and *paeX*), three beta-glucoside transport/utilisation operons (*bglHDJ, bglYK arbFBH*) and five di/tricarboxylate transporters (*dcuA*, *dcuB*, *dctA*, *maeN*, and *yflS*). For most of these genes, the PhoP binding site location suggests an activator role. At least one negative regulatory site (that could block PhoP-dependent transcription activation) could be located between PhoP operator and translation start site (attenuators in front of *bglH*, *bglY*, *arbF*, and possibly *paeX*, operators for DcuR in front of *dcuA, yflS*, and *dcuB*, operators for NarP in front of *dcuB* and *yflS*).

**FIGURE 7:**
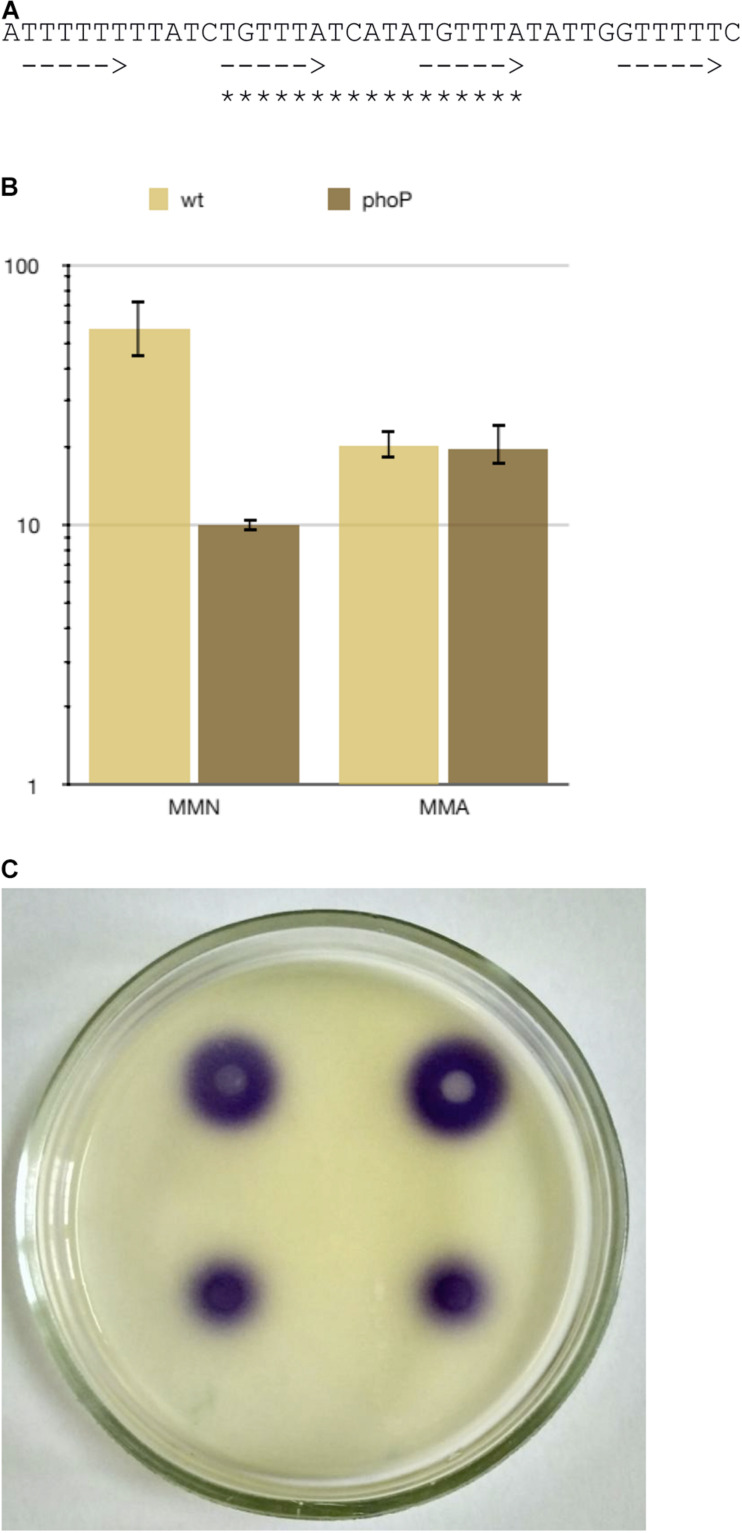
PhoP regulates quorum sensing via transcriptional control of the *expI* gene. **(A)** The sequence of the PhoP binding sites in *expI* promoter region. Arrows indicate possible positions of PhoP monomer binding sites, asterisks—the most likely position for binding of PhoP dimer. **(B)** *expI* expression in *Pve* cultures grown to 10^8^ cells ml^–1^ in MMN (10 μM Ca^2+^, 10 μM Mg^2+^) and MMA (10 μM Ca^2+^, 0.5 mM Mg^2+^). **(C)** *N-*acyl-homoserine lactone bioassay result. The purple colour indicates *N-*acyl-homoserine lactone-dependent violacein pigment production by the CV026 *C. violaceum* indicator strain. Top: the wild type strain, bottom: the *phoP* mutant.

A “strong” PhoP binding site is located in front of the *expI* gene coding for *N-*acyl-homoserine lactone synthase, a key regulator of quorum sensing. ExpI is known to dramatically affect gene expression, including that of many virulence genes (Liu et al., 2008), therefore PhoP control of *expI* transcription was studied in more detail. The regulatory region of *expI* (Figure 7A) has four equally spaced half-sites (two central ones perfectly match the gTTTA consensus) allowing for the binding of two PhoP dimers (Figure 7A). Since PhoP homologues can form oligomers (He et al., 2016), binding of a PhoP tetramer is also possible in this region.

The RNA-seq data showed a small (22%) difference in *expI* expression levels between the wild type and the mutant strains grown in MMA with 0.5 mM of magnesium ions (data not shown). However, a fivefold difference was observed if the bacteria were grown in the MMN medium at low concentrations of both calcium and magnesium ions when the PhoPQ system was fully induced (Figure 7B). Higher *expI* transcription in the wild type strain than in the mutant correlated with the higher production of *N-*acyl-homoserine lactone (Figure 7C). These results show an intriguing connection between the PhoPQ two-component and quorum-sensing systems in *Pve*.

Searching the NCBI nr database reveals that the nucleotide sequence very similar to the one shown in Figure 7A is present in many (although not all) *Pectobacterium* spp. genomes (data not shown), but could not be found outside the genus, making this particular connection between PhoPQ and the quorum sensing system *Pectobacterium*-specific.

Another regulatory region with multiple potential PhoP binding sites is located in front of the large *tss* operon coding for the subunits of the type VI secretion system (data not shown). We have not seen a noticeable effect of PhoP inactivation on downstream genes expression *in vitro.* However, recent work with *Pectobacterium brasiliense* has reported a strong PhoP-dependent induction of the *tss* genes in planta (Bellieny-Rabelo et al., 2020) suggesting that additional regulator(s) might block PhoP-dependent expression of the *tss* genes *in vitro* while this block is removed *in planta*.

The *expI* and *tss* gene examples confirm that at least some *in silico* predicted PhoP targets are true regulon members that can be controlled by PhoP in certain conditions. The list of *in silico* predicted PhoP regulon members (Supplementary File 2) might be helpful in further studies of regulatory networks and different aspects of *Pve* interaction with its environment, including host plants.

### PhoP Regulon Is Activated in *Pve* by Ca^2+^ and Mg^2+^ Limitation

**FIGURE 8:**
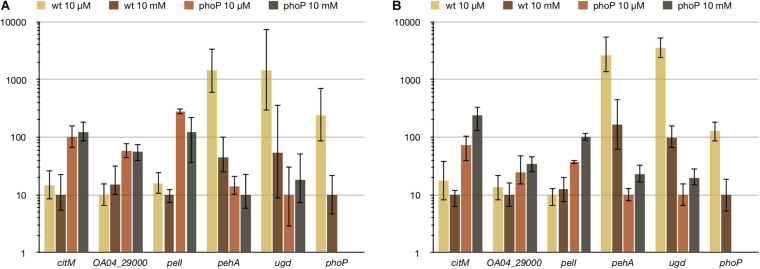
Ca^2+^ **(A)** and Mg^2+^ **(B)** reduce expression of PhoP-activated genes, but do not affect PhoP-repressed ones. For qPCR measurement, RNA was isolated from the wild type (wt) or *phoP* mutant *Pve* cells grown in MMN with either 10 μM or 10 mM of the specified ion. Mean values of relative expression levels with 95% confidence intervals are shown.

In an attempt to find the conditions responsible for PhoPQ activation in *Pve*, we checked some environmental stimuli known to be detected by the PhoQ sensor. There was no reaction to cationic peptide polymyxin and low pH (data not shown), but the response to divalent cations was significant. Expression levels of PhoP-activated genes were the highest at low (10 μM) concentrations of both Ca^2+^ and Mg^2+^ (Figure 8). Increasing either cation concentration to 10 mM reduced expression levels of PhoP-activated genes. Importantly, expression of *phoP* was decreased 13 times by 10 mM Mg^2+^ and 24 times by 10 mM Ca^2+^. Expression level differences of *pehA* and *ugd* caused by divalent cation addition were in the range 16–35 times. Comparison with the *phoP* mutant strain grown in the same conditions shows that some active PhoP must be present in the wild type cells even after divalent cation addition as *phoP* inactivation results in larger (91–353 times) drop of *pehA* and *ugd* expression levels. We interpret these results by both Mg^2+^ and Ca^2+^ being able to bind PhoQ to significantly reduce its kinase activity required for phosphorylation of PhoP resulting in a strong reduction of transcription initiation at PhoP-activated promoters.

Unexpectedly, divalent cation concentrations had little effect on PhoP-repressed genes. Expression levels of the three genes tested (*citM*, *pelI*, and *OA04_29000*) differed less than twofold between the wild type and *phoP* mutant strains and these differences were not statistically significant (Figure 8). We conclude that (i) PhoP amounts present in the wild type *Pve* cells grown at 10 mM Ca^2+^ or Mg^2+^ are sufficient to cause repression of the two transcriptional units involved and (ii) phosphorylation may not be required for PhoP to bind its operator sites. The last conclusion is in line with the previous report of both dimerisation and DNA binding of PhoP being independent of its phosphorylation (Perron-Savard et al., 2005). The ability of reduced PhoP amounts to repress transcription of *pelI* and the *citM-OA04_29000* operon is not surprising, as regulatory regions of both transcriptional units have multiple high scoring (and presumably high-affinity) PhoP binding sites.

### PhoP Regulon Expression *in planta*

Due to autoregulation, PhoP involvement in the adaptation to the plant environment must be accompanied by changes in its expression level. We have previously noticed decreased *phoP* expression in another example of *Pectobacterium*-plant interaction. A 3.5- and 5.2-fold *phoP* repression was observed for *P. atrosepticum* strain SCRI 1043 in asymptomatic and necrotic zones of infected tobacco stems (Gorshkov et al., 2018). This reduction of *phoP* expression correlated well with expression level changes of PhoP controlled genes: e.g., *pehA* expression was reduced while *pelI* and *ara* genes expression was increased.

**FIGURE 9:**
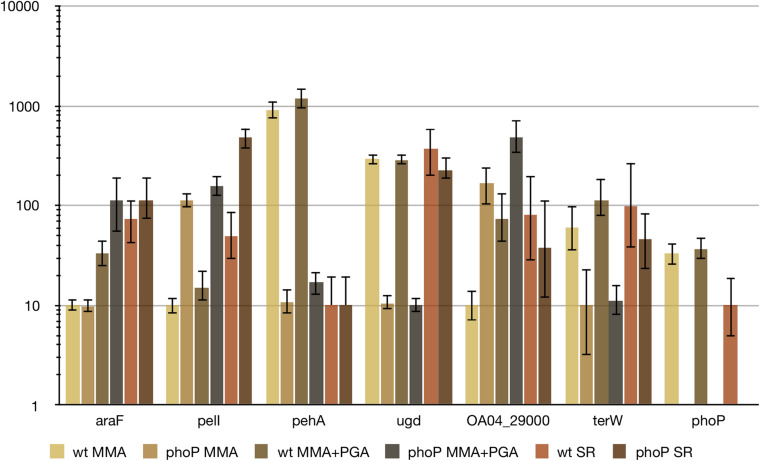
Relative expression levels of PhoP regulon members *in vitro* and *in planta.* For qPCR measurement, RNA was isolated from the wild type (wt) or *phoP* mutant *Pve* cells grown in MMA, MMA with 0.5% sodium polypectate (MMA + PGA) or from the cells of rotten potato tissues (SR).

In the current investigation, *phoP* expression decreased about 4 times in macerated potato tuber tissues (Figure 9). In the bacterial cells isolated from the rotten tuber tissues, expression level differences of PhoP regulon members between the wild type and mutant strains were much less pronounced than *in vitro* or even absent (Figure 9). This could be attributed to the partial inactivation of the PhoPQ system *in planta* as described in the Discussion section. A single notable exception was the *pelI* gene, which had similar differences of expression levels between the wild type and *phoP* mutant strains *in planta* and *in vitro*. A large number of high-affinity PhoP binding sites in front of *pelI* may permit even reduced amounts of PhoP to still cause *pelI* repression.

Two genes that show significantly higher expression in *phoP* mutant cells within potato tubers than *in vitro* are *pelI* and *ugd.* Regulatory regions of both genes contain binding sites for FNR, a key transcriptional activator under anaerobiosis. As the tubers were incubated in anaerobic conditions after inoculation, FNR-dependent transcription activation could be responsible for the observed higher *in planta* expression of *pelI* and *ugd*.

## Discussion

Our experimental results and *in silico* analysis show that plant pathogenesis-specific part constitutes the majority of PhoP-controlled genes in *Pve.* As a result, the PhoPQ two-component system can be considered a major modulator of *Pve* gene expression that ensures the levels of certain proteins are appropriate for particular stages of plant colonisation.

**FIGURE 10:**
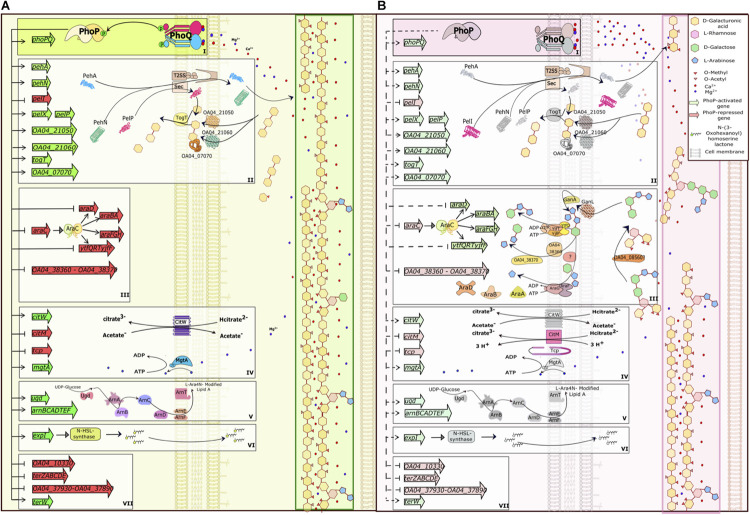
PhoPQ-dependent switch of virulence gene expression in *Pve*. **(A)** PhoQ kinase phosphorylates PhoP at low concentrations of calcium and magnesium ions (early infection) leading to maximal activation and repression of its targets. **(B)** As the infection progresses, extensive plant cell wall degradation releases vast amounts of Ca^2+^ and Mg^2+^ leading to inhibition of PhoQ kinase activity and PhoP dephosphorylation. This prevents PhoP-dependent activation, but still allows repression of target genes that have high-affinity PhoP operators. The drawing tries to link the expression of PhoPQ-controlled genes to plant cell wall degradation but does not take into the account other regulators that might influence the same genes. The picture shows the majority of PhoP-repressed genes and a selection of PhoP-activated genes from the same functional categories. The categories are framed and labelled I (the PhoPQ TCS), II (polygalacturonic acid degradation and utilisation), III (arabinogalactan degradation and utilisation), IV (transporters), V (LPS modification), VI (quorum sensing) and VII (tellurite resistance). In the categories II and III, the T2SS and Sec systems, two galactanases and the GanL porin not controlled by PhoP are added for clarity; these proteins are induced *in planta* and complete the full pathway of degradation, transport and utilisation of pectin components. To simplify the picture, categories IV and V are shown only partially; arabinogalactan degradation products are simplified (fewer sugar residues are drawn). Activation/repression are shown as pointed/blunt arrows. Activated/repressed genes are shaded green/red. PhoP-activated gene products are greyed on **(B)** to mark their presence in reduced amounts.

The major functional categories of PhoP-controlled proteins include stress resistance factors, hydrolases of plant cell wall polysaccharides, transporters and enzymes necessary for the utilisation of plant-derived products (Figure 10).

*phoP* transcription in *Pve* decreased within rotten tissues of potato tuber. We had previously seen a similar decrease in *phoP* transcription in a slightly different pathosystem (Gorshkov et al., 2018). In these two cases, the decrease of *phoP* transcription occurs at the late stages of infection. Recent work with *P. brasiliense* has not only reported decreased *phoP* expression at late infection stage but also registered an increase of *phoP* transcript amounts shortly after tuber inoculation (Bellieny-Rabelo et al., 2020). Since *phoP* is autoregulated, we think the changes of *phoP* expression level reflect the state of the PhoPQ two-component system: induced at the onset of the infection and repressed at later stages. The switch between the two states occurs due to the detection of plant-derived stimuli and helps the pathogen to adjust its properties, mostly cell envelope-related, that are required for proper interaction with the host plant.

Of the stimuli known to be detected by PhoQ in other bacteria, we were able to register strong response only to divalent cation concentrations. The pectobacterial PhoPQ system was originally described in *P. parmentieri* as responding to Ca^2+^ ions (Flego et al., 1997, 2000) while we have observed similar PhoPQ response to both Ca^2+^ and Mg^2+^ in *Pve*. In plant cells, Ca^2+^ was shown to be mostly cell wall-bound but it is released when the cell wall is degraded by pectobacteria (Park et al., 2004). Mg^2+^ concentration in plant apoplast was also reported to be quite low (Haque et al., 2008). On the other hand, the release of all ions bound in plant cells would result in concentrations of about 10 mM of Ca^2+^ and Mg^2+^ (Pagel and Heitefuss, 1989; Brown et al., 2012). Therefore, PhoPQ expression can be expected to be high at the onset of infection, resulting in the activation of the *pehA* gene expression. High polygalacturonase activity at the first stage of infection might change the availability of other cell wall components and would release cell wall-bound Ca^2+^. The combined action of divalent cations would lead to the repression of *phoPQ* transcription.

PelI is highly active in the presence of high Ca^2+^ concentrations, is known to be highly expressed *in planta* and is thought to be especially effective at the degradation of the calcium-rich middle lamella (Shevchik et al., 1997; Hugouvieux-Cotte-Pattat et al., 2014). High *pelI* expression, observed in potato tubers at late infection stages, may have a major effect on tissue maceration. Therefore, even higher PelI amounts in the *phoP* mutant can be the reason for its greater maceration ability during the artificial tuber inoculation.

The switch from PehA to PelI as the major pectinolytic enzyme correlates with the most likely sequence of events during plant cell wall polygalacturonate degradation and utilisation by a soft rot pathogen (Reverchon et al., 2016). Polygalacturonases have an acidic pH optimum and are therefore required at early stages of infection, as apoplastic fluid is slightly acidic. In contrast, PelI is active at the alkaline pH (Shevchik et al., 1997) and is relevant at the later stages of infection when the alkalinisation of the infection area occurs (Nachin and Barras, 2000). Release of further amounts of Ca^2+^ and Mg^2+^ by PelI may cause an even stronger decrease of *phoPQ* expression, resulting in the final stabilisation of PhoP regulon members expression at the levels required for the late stages of infection.

Contrary to PhoP-dependent transcription activation, repression does not seem to require phosphorylated PhoP and hence can still occur in the presence of high Ca^2+^ and/or Mg^2+^ concentrations, especially at promoter regions with multiple and/or high-affinity PhoP binding sites. However, as PhoP amounts are somewhat decreased in these conditions, the outcomes for different PhoP-repressed genes might vary: from partial derepression of genes with single and/or low-affinity PhoP binding site(s) to weak or almost no derepression of genes with multiple and/or high-affinity PhoP binding site(s).

An interesting aspect of PhoP-dependent repression links metabolic pathways of galacturonic acid-containing and arabinose-containing cell wall component utilisation. PhoP represses uptake and degradation of arabinose-containing cell wall components only in the presence of PGA. Although the exact mechanism of PGA involvement is not certain, the physiological consequences of such regulation are quite clear: galacturonic acid-containing cell wall breakdown products are preferred over arabinose-containing ones. This preference is relieved in the soft rot area (which could be due to exhausting PGA amounts) allowing the pathogen to utilise additional nutrients.

Many transporters are controlled by PhoP at least to some extent and most of them are activated. High transporter activity at the onset of infection would increase intracellular nutrient availability for the pathogen. Increased external nutrient availability at the later stages of infection justifies the reduction of transporter gene expression. Overall, transporter-related genes constitute approximately 1/3 of the DEGs with two largest groups of 7 genes each encoding iron and arabinose transporters. Citrate transporters seem to be a special case since one of them (CitM) is repressed while the other one (CitW) is activated by PhoP. The switch from CitW to CitM during plant colonisation may ensure continuous uptake of the abundant nutrient. Additional tri- and dicarboxylate transporters are likely to be controlled by PhoP too, but have been poorly expressed under conditions used in our work, possibly due to the missing inducers.

PhoP is also involved in the control of several stress resistance genes. The two largest groups of these genes defend *Pve* from cationic peptides and tellurite. Plants produce a rich arsenal of mostly CAMPs (Benko-Iseppon et al., 2010). The *ugd-arnBCADTEF* locus is strongly activated by PhoP and should protect against CAMPs from the early stages of infection when *Pve* cells may face high CAMP concentrations in the apoplast. In contrast, a large group of poorly characterised “tellurite resistance” genes are PhoP repressed and should be better expressed at the later stages of infection. PhoP-dependent coregulation of the *ter* genes with well-characterised virulence factors hints at their involvement in host adaptation. A possible contribution of these genes to oxidative resistance (Chasteen et al., 2009) may help plant pathogen to overcome host-generated oxidative burst. Since tellurite resistance genes are among the few that uniquely distinguish *Pve* from other pectobacteria, their detailed study may help to better understand the pathogenic strategy of this species.

It has long been established that anaerobic conditions are required for successful plant infection by soft rot bacteria (see Perombelon, 2002, for a review). This requirement was attributed to both the impairment of oxygen-dependent host resistance systems and increase of pathogen virulence. RNA-seq data show the involvement of PhoP in the regulation of 15 genes involved in anaerobic metabolism while *in silico* analysis suggests even more potential anaerobiosis-related PhoP targets. Since neither of these genes was reported to be controlled by PhoP in animal pathogens, their addition to PhoP regulon of *Pve* constitutes plant pathogen-specific extension of PhoP regulon. Some, but not all of the PhoP-controlled anaerobiosis-related genes are co-regulated by the main anaerobiosis regulator FNR, creating possibilities for complex regulatory effects. An interesting example is provided by cationic peptide resistance genes *ugd* and *arnBCADTEF*. Our results suggest that PhoP maintains a high expression level of these two transcription units in aerobic conditions *in vitro* while high expression level in *phoP* mutant cells within macerated potato tuber tissues can be explained by FNR-dependent transcription activation in anaerobic conditions. Other cases of *Pve*-specific PhoP and FNR operator co-occurrence include the *ter* operon and several pectinolysis genes.

Our results give additional hints to PhoPQ position in the regulatory network of *Pve*. Direct regulation of *expI* transcription links PhoP to the quorum sensing system. This link may result in a significant impact of PhoPQ on the secondary metabolism (including virulence factors production) via the well-established signal chain consisting of *N*-acylhomoserine lactone (AHL) produced by ExpI, AHL-responsive transcription factors ExpR (ExpR1) and VirR (ExpR2), and the global posttranscriptional regulator RsmA (Cui et al., 2006; Sjöblom et al., 2006). However, due to several autoregulatory mechanisms within the quorum sensing system and its intricate connections with other regulators, the exact outcome of increasing *expI* expression in a particular condition (especially *in planta*) is hard to predict. These mechanisms include autorepression of at least one of the AHL-responsive TFs (Castang et al., 2006) and interference between convergently transcribed *expI* and *expR* (Gogoleva et al., 2014). Additional complexity arises due to RsmA being targeted by multiple other regulators, including plant-responsive ones (Mukherjee et al., 2000; Hyytiainen et al., 2001). Of these, KdgR is the most important in the context of our work. KdgR directly represses transcription of RsmB, the regulatory RNA antagonising RsmA (Liu et al., 1999). In addition to this global regulatory mode, KdgR is also the major repressor of multiple pectinolysis genes (Liu et al., 1999). We have previously located KdgR binding sites within a pectobacterial genome (Nikolaichik and Damienikan, 2016) and now observed frequent co-occurrence of KdgR and PhoP binding sites in the regulatory regions of pectinolysis-related genes in *Pve*. This creates possibilities for delicate regulation of virulence properties, since both KdgR and PhoPQ can be inactivated *in planta* by their corresponding ligands, 2-keto-3-deoxygluconate (Nasser et al., 1992) and Ca^2+^/Mg^2+^. The mode of regulation is opposite for the two regulators (repression by KdgR and mostly activation by PhoP), hence the resulting changes in gene expression would depend on relative timing and extent of inactivation of the two regulators. Due to the overall complexity of the interaction network in soft rot pathogens, PhoP involvement in regulation of pectinolysis and anaerobiosis deserves dedicated studies.

We finally note that the PhoP regulon in *Pve* is likely to include many more genes apart from the DEGs found in this work. We believe that many of the additional PhoP binding sites found by the *in silico* approach are the true ones and can result in PhoP-dependent expression level changes in conditions different from the ones studied here. Taking into account numerous potential PhoP targets, high proportion of virulence-related and regulatory genes among them and likely interactions with other regulators, the possibility of PhoP involvement should be routinely considered when studying the expression of virulence factors in *Pve* and related plant pathogens.

## Data Availability Statement

The datasets presented in this study can be found in online repositories. The names of the repository/repositories and accession number(s) can be found below: https://www.ncbi.nlm.nih.gov/genbank/, PRJNA627079.

## Author Contributions

UK: phoP mutant construction and preliminary characterisation, assessment of polymyxin B and tellurite resistance, virulence tests, gene expression measurement with qRT-PCR, visualisation, and writing, reviewing, and editing of the manuscript. NG: cDNA library preparation, sequencing, data quality control, and filtering. NK: gene expression measurement with qRT-PCR. AK: RNA-seq data analysis. YD: construction of the pPQ plasmid and complementation tests. YG: conceptualisation of the study, funding acquisition, resources, supervision, and reviewing and editing of the manuscript. YN: conceptualisation of the study, funding acquisition, resources, supervision, writing, reviewing, and editing of the manuscript, RNA isolation, virulence tests, structure analysis, TFBS inference, PhoP regulon analysis, and genome annotation correction. All authors contributed to the article and approved the submitted version.

## Conflict of Interest

The authors declare that the research was conducted in the absence of any commercial or financial relationships that could be construed as a potential conflict of interest.

## Footnotes

1 https://github.com/nikolaichik/SigmoID

## References

[B1] Abbott D. W. Boraston A. B. (2008). Structural biology of pectin degradation by *Enterobacteriaceae*. *Microbiol. Mol. Biol. Rev.* 72 301–316. 10.1128/MMBR.00038-07 18535148 PMC2415742

[B2] Abbott D. W. Hrynuik S. Boraston A. B. (2007). Identification and characterization of a novel periplasmic polygalacturonic acid binding protein from *Yersinia enterolitica*. *J. Mol. Biol.* 367 1023–1033. 10.1016/j.jmb.2007.01.030 17292916

[B3] Anantharaman V. Iyer L. M. Aravind L. (2012). Ter-dependent stress response systems: novel pathways related to metal sensing, production of a nucleoside-like metabolite, and DNA-processing. *Mol. Biosyst.* 8 3142–3165. 10.1039/c2mb25239b 23044854 PMC4104200

[B4] Bader M. W. Sanowar S. Daley M. E. Schneider A. R. Cho U. Xu W. (2005). Recognition of antimicrobial peptides by a bacterial sensor kinase. *Cell* 122 461–472. 10.1016/j.cell.2005.05.030 16096064

[B5] Bellieny-Rabelo D. Nkomo N. P. Shyntum D. Y. Moleleki L. N. (2020). Horizontally acquired quorum-sensing regulators recruited by the phop regulatory network expand the host adaptation repertoire in the phytopathogen *Pectobacterium brasiliense*. *mSystems* 5 e619–e650. 10.1128/mSystems.00650-19 31992632 PMC6989131

[B6] Benko-Iseppon A. M. Lins Galdino S. Calsa T. Jr. Akio Kido E. Tossi A. Carlos Belarmino L. (2010). Overview on plant antimicrobial peptides. *Curr. Protein Pept. Sci.* 11 181–188. 10.2174/138920310791112075 20088772

[B7] Bera A. Herbert S. Jakob A. Vollmer W. Götz F. (2004). Why are pathogenic staphylococci so lysozyme resistant? the peptidoglycan O-acetyltransferase OatA is the major determinant for lysozyme resistance of Staphylococcus aureus: lysozyme resistance of S. aureus. *Mol. Microbiol.* 55 778–787. 10.1111/j.1365-2958.2004.04446.x 15661003

[B8] Berman H. M. (2000). The protein data bank. *Nucleic Acids Res.* 28 235–242. 10.1093/nar/28.1.235 10592235 PMC102472

[B9] Bott M. (1997). Anaerobic citrate metabolism and its regulation in enterobacteria. *Arch. Microbiol.* 167 78–88. 10.1007/s002030050419 9133329

[B10] Bowman S. E. J. Backman L. R. F. Bjork R. E. Andorfer M. C. Yori S. Caruso A. (2019). Solution structure and biochemical characterization of a spare part protein that restores activity to an oxygen-damaged glycyl radical enzyme. *J. Biol. Inorg. Chem.* 24 817–829. 10.1007/s00775-019-01681-2 31250200 PMC6754787

[B11] Bray N. L. Pimentel H. Melsted P. Pachter L. (2016). Near-optimal probabilistic RNA-seq quantification. *Nat. Biotechnol.* 34 525–527. 10.1038/nbt.3519 27043002

[B12] Brown C. R. Haynes K. G. Moore M. Pavek M. J. Hane D. C. Love S. L. (2012). Stability and broad-sense heritability of mineral content in potato: calcium and magnesium. *Am. J. Potato Res.* 89 255–261. 10.1007/s12230-012-9240-9

[B13] Bullock W. O. (1987). XL1-Blue: a high efficiency plasmid transforming recA *Escherichia coli* strain with beta-galactosidase selection. *Bio. Tecch.* 5 376–379.

[B14] Burr T. Barnard A. M. L. Corbett M. J. Pemberton C. L. Simpson N. J. L. Salmond G. P. C. (2006). Identification of the central quorum sensing regulator of virulence in the enteric phytopathogen, erwinia carotovora: the VirR repressor. *Mol. Microbiol.* 59 113–125. 10.1111/j.1365-2958.2005.04939.x 16359322

[B15] Castang S. Reverchon S. Gouet P. Nasser W. (2006). Direct evidence for the modulation of the activity of the erwinia chrysanthemi quorum-sensing regulator ExpR by acylhomoserine lactone pheromone. *J. Biol. Chem.* 281 29972–29987. 10.1074/jbc.M601666200 16831870

[B16] Chasteen T. G. Fuentes D. E. Tantaleán J. C. Vásquez C. C. (2009). Tellurite: history, oxidative stress, and molecular mechanisms of resistance. *FEMS Microbiol. Rev.* 33 820–832. 10.1111/j.1574-6976.2009.00177.x 19368559

[B17] Chatterjee A. Cui Y. Chaudhuri S. Chatterjee A. K. (2002). Identification of regulators of hrp/hop genes of Erwinia carotovora ssp carotovora and characterization of HrpLEcc (SigmaLEcc), an alternative sigma factor. *Mol. Plant Pathol.* 3 359–370. 10.1046/j.1364-3703.2002.00128.x 20569343

[B18] Coornaert A. Chiaruttini C. Springer M. Guillier M. (2013). Post-transcriptional control of the *Escherichia coli* PhoQ-PhoP two-component system by multiple sRNAs involves a novel pairing region of GcvB. *PLoS Genet.* 9:e1003156. 10.1371/journal.pgen.1003156 23300478 PMC3536696

[B19] Cromie M. J. Groisman E. A. (2010). Promoter and Riboswitch Control of the Mg2+ Transporter MgtA from *Salmonella enterica*. *J. Bacteriol.* 192 604–607. 10.1128/JB.01239-09 19897653 PMC2805325

[B20] Cui Y. Chatterjee A. Chatterjee A. K. (2001). Effects of the two-component system comprising GacA and GacS of Erwinia carotovora subsp. carotovora on the production of global regulatory rsmB RNA, extracellular enzymes, and harpinEcc. *Mol. Plant Microbe. Interact.* 14 516–526. 10.1094/mpmi.2001.14.4.516 11310739

[B21] Cui Y. Chatterjee A. Hasegawa H. Chatterjee A. K. (2006). Erwinia carotovora subspecies produce duplicate variants of ExpR, LuxR homologs that activate rsmA transcription but differ in their interactions with N-acylhomoserine lactone signals. *J. Bacteriol.* 188 4715–4726. 10.1128/JB.00351-06 16788181 PMC1483022

[B22] Cui Y. Chatterjee A. Hasegawa H. Dixit V. Leigh N. Chatterjee A. K. (2005). ExpR, a LuxR homolog of Erwinia carotovora subsp. carotovora, activates transcription of rsmA, which specifies a global regulatory RNA-binding protein. *J. Bacteriol.* 187 4792–4803. 10.1128/JB.187.14.4792-4803.2005 15995194 PMC1169500

[B23] Cui Y. Chatterjee A. Yang H. Chatterjee A. K. (2008). Regulatory network controlling extracellular proteins in erwinia carotovora subsp. carotovora: FlhDC, the master regulator of flagellar genes, activates rsmb regulatory RNA production by affecting gacA and hexA (lrhA) expression. *J. Bacteriol.* 190 4610–4623. 10.1128/JB.01828-07 18441056 PMC2446818

[B24] Delic-Attree I. Toussaint B. Garin J. Vignais P. M. (1997). Cloning, sequence and mutagenesis of the structural gene of *Pseudomonas aeruginosa* CysB, which can activate *algD* transcription. *Mol. Microbiol.* 24 1275–1284. 10.1046/j.1365-2958.1997.4121799.x 9218775

[B25] Eddy S. R. (2011). Accelerated profile HMM searches. *PLoS Comput. Biol.* 7:e1002195. 10.1371/journal.pcbi.1002195 22039361 PMC3197634

[B26] Farrow J. M. Hudson L. L. Wells G. Coleman J. P. Pesci E. C. (2015). CysB negatively affects the transcription of *pqsR* and *Pseudomonas* quinolone signal production in *Pseudomonas aeruginosa*. *J. Bacteriol.* 197 1988–2002. 10.1128/JB.00246-15 25845844 PMC4438213

[B27] Flego D. Marits R. Eriksson A. R. Koiv V. Karlsson M. B. Heikinheimo R. (2000). A two-component regulatory system, pehR-pehS, controls endopolygalacturonase production and virulence in the plant pathogen erwinia carotovora subsp. carotovora. *Mol. Plant Microbe. Interact.* 13 447–455. 10.1094/mpmi.2000.13.4.447 10755308

[B28] Flego D. Pirhonen M. Saarilahti H. Palva T. K. Palva E. T. (1997). Control of virulence gene expression by plant calcium in the phytopathogen Erwinia carotovora. *Mol. Microbiol.* 25 831–838. 10.1111/j.1365-2958.1997.mmi501.x 9364909

[B29] Fujimoto Z. Ichinose H. Maehara T. Honda M. Kitaoka M. Kaneko S. (2010). Crystal Structure of an Exo-1,5-α-l-arabinofuranosidase from *Streptomyces avermitilis* provides insights into the mechanism of substrate discrimination between Exo- and Endo-type Enzymes in glycoside hydrolase family 43. *J. Biol. Chem.* 285 34134–34143. 10.1074/jbc.M110.164251 20739278 PMC2962512

[B30] Garriga X. Eliasson R. Torrents E. Jordan A. Barbé J. Gibert I. (1996). nrdD and nrdG genes are essential for strict anaerobic growth of *Escherichia coli*. *Biochem. Biophys. Res. Commun.* 229 189–192. 10.1006/bbrc.1996.1778 8954104

[B31] Gogoleva N. Kravchenko U. Nikolaichik Y. Gogolev Y. (2020). Transcriptomic dataset of wild type and phoP mutant *Pectobacterium* versatile. *Data Brief* 32:106123. 10.1016/j.dib.2020.106123 32817874 PMC7424204

[B32] Gogoleva N. E. Shlykova L. V. Gorshkov V. Y. Daminova A. G. Gogolev Y. V. (2014). Effect of topology of quorum sensing-related genes in *Pectobacterium* atrosepticum on their expression. *Mol. Biol.* 48 583–589. 10.1134/S0026893314040049 25842850

[B33] Gorshkov V. Gubaev R. Petrova O. Daminova A. Gogoleva N. Ageeva M. (2018). Transcriptome profiling helps to identify potential and true molecular switches of stealth to brute force behavior in Pectobacterium atrosepticum during systemic colonization of tobacco plants. *Eur. J. Plant Pathol.* 152 957–976. 10.1007/s10658-018-1496-6

[B34] Groisman E. A. Hollands K. Kriner M. A. Lee E.-J. Park S.-Y. Pontes M. H. (2013). Bacterial Mg ^2+^ homeostasis, transport, and virulence. *Annu. Rev. Genet.* 47 625–646. 10.1146/annurev-genet-051313-051025 24079267 PMC4059682

[B35] Haque M. M. Nahar K. Rahim M. A. Gomes I. Tsuyumu S. (2008). PhoP-PhoQ Two-component system required for colonization leading to virulence of dickeya dadantii 3937 in planta. *Bangladesh J. Microbiol.* 25 36–40. 10.3329/bjm.v25i1.4853

[B36] Haque M. M. Tsuyumu S. (2005). Virulence, resistance to magainin II, and expression of pectate lyase are controlled by the PhoP-PhoQ two-component regulatory system responding to pH and magnesium in Erwinia chrysanthemi 3937. *J. Gen. Plant Pathol.* 71 47–53. 10.1007/s10327-004-0158-z

[B37] Harari O. Park S.-Y. Huang H. Groisman E. A. Zwir I. (2010). Defining the plasticity of transcription factor binding sites by deconstructing DNA consensus sequences: the PhoP-binding sites among gamma/enterobacteria. *PLoS Comput. Biol.* 6:e1000862. 10.1371/journal.pcbi.1000862 20661307 PMC2908699

[B38] Harris S. J. Shih Y. L. Bentley S. D. Salmond G. P. (1998). The hexA gene of Erwinia carotovora encodes a LysR homologue and regulates motility and the expression of multiple virulence determinants. *Mol. Microbiol.* 28 705–717. 10.1046/j.1365-2958.1998.00825.x 9643539

[B39] He X. Wang L. Wang S. (2016). Structural basis of DNA sequence recognition by the response regulator PhoP in Mycobacterium tuberculosis. *Sci. Rep.* 6:24442. 10.1038/srep24442 27079268 PMC4832192

[B40] Horler R. S. P. Müller A. Williamson D. C. Potts J. R. Wilson K. S. Thomas G. H. (2009). Furanose-specific sugar transport characterization of a bacterial galactofuranose-binding protein. *J. Biol. Chem.* 284 31156–31163. 10.1074/jbc.M109.054296 19744923 PMC2781514

[B41] Hugouvieux-Cotte-Pattat N. Condemine G. Shevchik V. E. (2014). Bacterial pectate lyases, structural and functional diversity: bacterial pectate lyases. *Environ. Microbiol. Rep.* 6 427–440. 10.1111/1758-2229.12166 25646533

[B42] Hyytiainen H. Montesano M. Palva E. T. (2001). Global regulators ExpA (GacA) and KdgR modulate extracellular enzyme gene expression through the RsmA-rsmB system in Erwinia carotovora subsp. carotovora. *Mol. Plant Microbe. Interact.* 14 931–938. 10.1094/mpmi.2001.14.8.931 11497464

[B43] Imperi F. Tiburzi F. Fimia G. M. Visca P. (2010). Transcriptional control of the *pvdS* iron starvation sigma factor gene by the master regulator of sulfur metabolism CysB in *Pseudomonas aeruginosa*. *Environ. Microbiol.* 12 1630–1642. 10.1111/j.1462-2920.2010.02210.x 20370820

[B44] Iwadate Y. Kato J. (2019). Identification of a formate-dependent uric acid degradation pathway in *Escherichia coli*. *J. Bacteriol.* 201 e518–e573. 10.1128/JB.00573-18 30885932 PMC6509651

[B45] Jafra S. Figura I. Hugouvieux-Cotte-Pattat N. Lojkowska E. (1999). Expression of erwinia chrysanthemi pectinase genes pelI, pelL, and pelZ during infection of potato tubers. *Mol. Plant-Microbe. Interact.* 12 845–851. 10.1094/mpmi.1999.12.10.845

[B46] Kästner C. N. Schneider K. Dimroth P. Pos K. M. (2002). Characterization of the citrate/acetate antiporter CitW of *Klebsiella pneumoniae*. *Arch. Microbiol.* 177 500–506. 10.1007/s00203-002-0420-8 12029396

[B47] Kotake T. Yamanashi Y. Imaizumi C. Tsumuraya Y. (2016). Metabolism of L-arabinose in plants. *J. Plant Res.* 129 781–792. 10.1007/s10265-016-0834-z 27220955 PMC5897480

[B48] Kravchenko U. Gogoleva N. Kolubako A. Kruk A. Diubo J. Gogolev Y. (2020). The PhoPQ two-component system is the major regulator of cell surface properties, stress responses and plant-derived substrate utilisation during development of *Pectobacterium versatile* -host plant pathosystem. *bioRxiv* 2020:060806 10.1101/2020.04.24.060806 PMC7843439 33519782

[B49] Kulakovskiy I. V. Boeva V. A. Favorov A. V. Makeev V. J. (2010). Deep and wide digging for binding motifs in ChIP-Seq data. *Bioinforma. Oxf. Engl.* 26 2622–2623. 10.1093/bioinformatics/btq488 20736340

[B50] Linares-Pastén J. A. Falck P. Albasri K. Kjellström S. Adlercreutz P. Logan D. T. (2017). Three-dimensional structures and functional studies of two GH43 arabinofuranosidases from *Weissella* sp. strain 142 and *Lactobacillus brevis*. *FEBS J.* 284 2019–2036. 10.1111/febs.14101 28485897

[B51] Liu H. Coulthurst S. J. Pritchard L. Hedley P. E. Ravensdale M. Humphris S. (2008). Quorum sensing coordinates brute force and stealth modes of infection in the plant pathogen *Pectobacterium* atrosepticum. *PLoS Pathog.* 4:e1000093. 10.1371/journal.ppat.1000093 18566662 PMC2413422

[B52] Liu Y. Chatterjee A. Chatterjee A. K. (1994). Nucleotide sequence, organization and expression of rdgA and rdgB genes that regulate pectin lyase production in the plant pathogenic bacterium Erwinia carotovora subsp. carotovora in response to DNA-damaging agents. *Mol. Microbiol.* 14 999–1010. 10.1111/j.1365-2958.1994.tb01334.x 7715460

[B53] Liu Y. Jiang G. Cui Y. Mukherjee A. Ma W. L. Chatterjee A. K. (1999). KdgREcc negatively regulates genes for pectinases, cellulase, protease, HarpinEcc, and a global RNA regulator in Erwinia carotovora subsp. carotovora. *J. Bacteriol.* 181 2411–2421. 10.1128/jb.181.8.2411-2421.1999 10198003 PMC93665

[B54] Lombard V. Golaconda Ramulu H. Drula E. Coutinho P. M. Henrissat B. (2014). The carbohydrate-active enzymes database (CAZy) in 2013. *Nucleic Acids Res.* 42 D490–D495. 10.1093/nar/gkt1178 24270786 PMC3965031

[B55] Manjurul Haque M. Hirata H. Tsuyumu S. (2012). Role of PhoP–PhoQ two-component system in pellicle formation, virulence and survival in harsh environments of Dickeya dadantii 3937. *J. Gen. Plant Pathol.* 78 176–189. 10.1007/s10327-012-0372-z

[B56] Matsuo N. Kaneko S. Kuno A. Kobayashi H. Kusakabe I. (2000). Purification, characterization and gene cloning of two α-L-arabinofuranosidases from Streptomyces chartreusis GS901. *Biochem. J.* 346 9–15. 10.1042/bj3460009 10657233 PMC1220816

[B57] McClean K. H. Chhabra S. R. Camara M. Daykin M. Swift S. Bycroft B. W. (1997). Quorum sensing and *Chromobacteriurn* violaceum:exploitation of violacein production and inhibition for the detection of N-acylhomoserine lactones. *Microbiology* 143 3703–3711. 10.1099/00221287-143-12-3703 9421896

[B58] McClure R. Balasubramanian D. Sun Y. Bobrovskyy M. Sumby P. Genco C. A. (2013). Computational analysis of bacterial RNA-Seq data. *Nucleic Acids Res.* 41:e140. 10.1093/nar/gkt444 23716638 PMC3737546

[B59] Metcalf W. W. Jiang W. Wanner B. L. (1994). Use of the rep technique for allele replacement to construct new *Escherichia coli* hosts for maintenance of R6K gamma origin plasmids at different copy numbers. *Gene* 138 1–7. 10.1016/0378-1119(94)90776-5 8125283

[B60] Meyer S. De Angeli A. Fernie A. R. Martinoia E. (2010). Intra- and extra-cellular excretion of carboxylates. *Trends Plant Sci.* 15 40–47. 10.1016/j.tplants.2009.10.002 19913451

[B61] Moreira C. G. Herrera C. M. Needham B. D. Parker C. T. Libby S. J. Fang F. C. (2013). Virulence and stress-related periplasmic protein (VisP) in bacterial/host associations. *Proc. Natl. Acad. Sci. U S A.* 110 1470–1475. 10.1073/pnas.1215416110 23302685 PMC3557018

[B62] Mukherjee A. Cui Y. Ma W. Liu Y. Chatterjee A. K. (2000). hexA of Erwinia carotovora ssp. carotovora strain Ecc71 negatively regulates production of RpoS and rsmB RNA, a global regulator of extracellular proteins, plant virulence and the quorum-sensing signal, N-(3-oxohexanoyl)- l-homoserine lactone. *Environ. Microbiol.* 2 203–215. 10.1046/j.1462-2920.2000.00093.x 11220306

[B63] Myamin V. E. Pesnyakevich A. G. Nikolaichik E. A. Prokulevich V. A. (2004). Genetic regulation of pathogenicity and virulence factors in bacteria erwinia carotovora subsp. atroseptica: identification of gene kduD. *Russ. J. Genet.* 40 970–976. 10.1023/B:RUGE.0000041374.14133.20 15559145

[B64] Nachin L. Barras F. (2000). External pH: an environmental signal that helps to rationalize pel gene duplication in Erwinia chrysanthemi. *Mol. Plant Microbe. Interact.* 6 882–886. 10.1094/mpmi.2000.13.8.882 10939260

[B65] Nasser W. Reverchon S. Robert-Baudouy J. (1992). Purification and functional characterization of the KdgR protein, a major repressor of pectinolysis genes of Erwinia chrysanthemi. *Mol. Microbiol.* 65 257–265. 10.1111/j.1365-2958.1992.tb02007.x 1545709

[B66] Needham B. D. Trent M. S. (2013). Fortifying the barrier: the impact of lipid A remodelling on bacterial pathogenesis. *Nat. Rev. Microbiol.* 11 467–481. 10.1038/nrmicro3047 23748343 PMC6913092

[B67] Nelson D. L. Kennedy E. P. (1971). Magnesium transport in *Escherichia coli*. Inhibition by cobaltous ion. *J. Biol. Chem.* 246 3042–3049. 4928897

[B68] Nikolaichik Y. Damienikan A. U. (2016). SigmoID: a user-friendly tool for improving bacterial genome annotation through analysis of transcription control signals. *PeerJ.* 4:e2056. 10.7717/peerj.2056 27257541 PMC4888284

[B69] Novichkov P. S. Kazakov A. E. Ravcheev D. A. Leyn S. A. Kovaleva G. Y. Sutormin R. A. (2013). RegPrecise 3.0 – A resource for genome-scale exploration of transcriptional regulation in bacteria. *BMC Genom.* 14:745. 10.1186/1471-2164-14-745 24175918 PMC3840689

[B70] Pagel W. Heitefuss R. (1989). Calcium content and cell wall polygalacturonans in potato tubers of cultivars with different susceptibilities to Erwinia carotovora subsp. atroseptica. *Physiol. Mol. Plant Pathol.* 35 11–21. 10.1016/0885-5765(89)90003-9

[B71] Park S.-W. Hwang B.-H. Kim W.-Y. Kim J. (2004). Changes in cell wall carbohydrates composition and Ca distribution of Brassica campestris ssp. pekinesis in relation to Erwinia polygalacturonase production during soft rot development. *Hortic. Environ. Biotechnol.* 45 223–232.

[B72] Pelosi L. Vo C.-D.-T. Abby S. S. Loiseau L. Rascalou B. Hajj Chehade M. (2019). Ubiquinone biosynthesis over the entire O _2_ range: characterization of a conserved O _2_ -independent pathway. *mBio* 10:e1319. 10.1128/mBio.01319-19 31289180 PMC6747719

[B73] Penfold R. J. Pemberton J. M. (1992). An improved suicide vector for construction of chromosomal insertion mutations in bacteria. *Gene* 118 145–146. 10.1016/0378-1119(92)90263-o 1511879

[B74] Perez J. C. Shin D. Zwir I. Latifi T. Hadley T. J. Groisman E. A. (2009). Evolution of a bacterial regulon controlling virulence and Mg2+ homeostasis. *PLoS Genet.* 5:1000428. 10.1371/journal.pgen.1000428 19300486 PMC2650801

[B75] Perombelon M. C. M. (2002). Potato diseases caused by soft rot erwinias: an overview of pathogenesis. *Plant Pathol.* 51 1–12. 10.1046/j.0032-0862.2001.short

[B76] Perron-Savard P. De Crescenzo G. Moual H. L. (2005). Dimerization and DNA binding of the *Salmonella enterica* PhoP response regulator are phosphorylation independent. *Microbiology* 151 3979–3987. 10.1099/mic.0.28236-0 16339942

[B77] Pfaffl M. W. Horgan G. W. Dempfle L. (2002). Relative expression software tool (REST©) for group-wise comparison and statistical analysis of relative expression results in real-time PCR. *Nucleic Acids Res.* 30:e36. 10.1093/nar/30.9.e36 11972351 PMC113859

[B78] Portier P. Pédron J. Taghouti G. Fischer-Le Saux M. Caullireau E. Bertrand C. (2019). Elevation of pectobacterium carotovorum subsp. odoriferum to species level as pectobacterium odoriferum sp. nov., proposal of pectobacterium brasiliense sp. nov. and pectobacterium actinidiae sp. nov., emended description of pectobacterium carotovorum and description of pectobacterium versatile sp. nov., isolated from streams and symptoms on diverse plants. *Int. J. Syst. Evol. Microbiol.* 69 3207–3216. 10.1099/ijsem.0.003611 31343401

[B79] Prost L. R. Daley M. E. Le Sage V. Bader M. W. Le Moual H. Klevit R. E. (2007). Activation of the bacterial sensor kinase PhoQ by acidic pH. *Mol. Cell* 26 165–174. 10.1016/j.molcel.2007.03.008 17466620

[B80] Raghavan R. Groisman E. A. Ochman H. (2011). Genome-wide detection of novel regulatory RNAs in E. coli. *Genome Res.* 21 1487–1497. 10.1101/gr.119370.110 21665928 PMC3166833

[B81] Reverchon S. Muskhelisvili G. Nasser W. (2016). *“Virulence Program of a Bacterial Plant Pathogen: The Dickeya Model,” in Progress in Molecular Biology and Translational Science.* Amsterdam: Elsevier 51–92. 10.1016/bs.pmbts.2016.05.005 27571692

[B82] Robinson M. D. McCarthy D. J. Smyth G. K. (2010). edgeR: a Bioconductor package for differential expression analysis of digital gene expression data. *Bioinformatics* 26 139–140. 10.1093/bioinformatics/btp616 19910308 PMC2796818

[B83] Sahota G. Stormo G. D. (2010). Novel sequence-based method for identifying transcription factor binding sites in prokaryotic genomes. *Bioinformatics* 26 2672–2677. 10.1093/bioinformatics/btq501 20807838 PMC2981494

[B84] Schleif R. (2010). AraC protein, regulation of the l-arabinose operon in *Escherichia coli*, and the light switch mechanism of AraC action. *FEMS Microbiol. Rev.* 34 779–796. 10.1111/j.1574-6976.2010.00226.x 20491933

[B85] Sharma C. M. Papenfort K. Pernitzsch S. R. Mollenkopf H.-J. Hinton J. C. D. Vogel J. (2011). Pervasive post-transcriptional control of genes involved in amino acid metabolism by the Hfq-dependent GcvB small RNA: GcvB regulon. *Mol. Microbiol.* 81 1144–1165. 10.1111/j.1365-2958.2011.07751.x 21696468

[B86] Shevchik V. E. Boccara M. Vedel R. Hugouvieux-Cotte-Pattat N. (1998). Processing of the pectate lyase PelI by extracellular proteases of Erwinia chrysanthemi 3937. *Mol. Microbiol.* 29 1459–1469. 9781882 10.1046/j.1365-2958.1998.01028.x

[B87] Shevchik V. E. Robert-Baudouy J. Hugouvieux-Cotte-Pattat N. (1997). Pectate lyase PelI of Erwinia chrysanthemi 3937 belongs to a new family. *J. Bacteriol.* 179 7321–7330. 10.1128/jb.179.23.7321-7330.1997 9393696 PMC179682

[B88] Shirshikov F. V. Korzhenkov A. A. Miroshnikov K. K. Kabanova A. P. Barannik A. P. Ignatov A. N. (2018). Draft genome sequences of new genomospecies “Candidatus Pectobacterium maceratum” strains, which cause soft rot in plants. *Genome Announc.* 6 e218–e260. 10.1128/genomeA.00260-18 29650577 PMC5897815

[B89] Sjöblom S. Brader G. Koch G. Palva E. T. (2006). Cooperation of two distinct ExpR regulators controls quorum sensing specificity and virulence in the plant pathogen Erwinia carotovora. *Mol. Microbiol.* 60 1474–1489. 10.1111/j.1365-2958.2006.05210.x 16796682

[B90] Slauch J. M. Lee A. A. Mahan M. J. Mekalanos J. J. (1996). Molecular characterization of the oafA locus responsible for acetylation of *Salmonella* typhimurium O-antigen: oafA is a member of a family of integral membrane trans-acylases. *J. Bacteriol.* 178 5904–5909. 10.1128/jb.178.20.5904-5909.1996 8830685 PMC178445

[B91] Stringer A. M. Currenti S. Bonocora R. P. Baranowski C. Petrone B. L. Palumbo M. J. (2014). Genome-scale analyses of *Escherichia coli* and *Salmonella enterica* AraC reveal noncanonical targets and an expanded core regulon. *J. Bacteriol.* 196 660–671. 10.1128/JB.01007-13 24272778 PMC3911152

[B92] Thomson N. R. Nasser W. McGowan S. Sebaihia M. Salmond G. P. (1999). Erwinia carotovora has two KdgR-like proteins belonging to the IciR family of transcriptional regulators: identification and characterization of the RexZ activator and the KdgR repressor of pathogenesis. *Microbiology* 145 1531–1545. 10.1099/13500872-145-7-1531 10439393

[B93] Toth I. K. Birch P. R. (2005). Rotting softly and stealthily. *Curr. Opin. Plant Biol.* 8 424–429. 10.1016/j.pbi.2005.04.001 15970273

[B94] Touzé T. Blanot D. Mengin-Lecreulx D. (2008a). Substrate specificity and membrane topology of *Escherichia coli* PgpB, an undecaprenyl pyrophosphate phosphatase. *J. Biol. Chem.* 283 16573–16583. 10.1074/jbc.M800394200 18411271

[B95] Touzé T. Tran A. X. Hankins J. V. Mengin-Lecreulx D. Trent M. S. (2008b). Periplasmic phosphorylation of lipid a is linked to the synthesis of undecaprenyl phosphate. *Mol. Microbiol.* 67 264–277. 10.1111/j.1365-2958.2007.06044.x 18047581 PMC2229476

[B96] Tsers I. Gorshkov V. Gogoleva N. Parfirova O. Petrova O. Gogolev Y. (2020). Plant soft rot development and regulation from the viewpoint of transcriptomic profiling. *Plants* 9:1176. 10.3390/plants9091176 32927917 PMC7570247

[B97] Untergasser A. Cutcutache I. Koressaar T. Ye J. Faircloth B. C. Remm M. (2012). Primer3—new capabilities and interfaces. *Nucleic Acids Res.* 40:e115. 10.1093/nar/gks596 22730293 PMC3424584

[B98] Urbany C. Neuhaus H. E. (2008). Citrate uptake into pectobacterium atrosepticum is critical for bacterial virulence. *Mol. Plant. Microbe Interact.* 21 547–554. 10.1094/MPMI-21-5-0547 18393614

[B99] Valková D. Valkovičová L. Vávrová S. Kováčová E. Mravec J. Turňa J. (2007). The contribution of tellurite resistance genes to the fitness of *Escherichia coli* uropathogenic strains. *Open Life Sci.* 2 182–191. 10.2478/s11535-007-0019-9

[B100] Valkovicova L. Valkova D. Vavrova S. Alekhina O. Hoang V. P. Jezna M. (2011). The role of TerW protein in the tellurite resistance of uropathogenic *Escherichia coli*. *Biologia* 66 565–573. 10.2478/s11756-011-0075-5

[B101] Vandesompele J. De Preter K. Pattyn F. Poppe B. Van Roy N. De Paepe A. (2002). Accurate normalization of real-time quantitative RT-PCR data by geometric averaging of multiple internal control genes. *Genome Biol.* 3:34. 10.1186/gb-2002-3-7-research0034 PMC126239 12184808

[B102] Véscovi E. G. Soncini F. C. Groisman E. A. (1996). Mg2+ as an Extracellular Signal: Environmental Regulation of *Salmonella* Virulence. *Cell* 84 165–174. 10.1016/S0092-8674(00)81003-X 8548821

[B103] Wagner A. F. V. Schultz S. Bomke J. Pils T. Lehmann W. D. Knappe J. (2001). YfiD of *Escherichia coli* and Y06I of Bacteriophage T4 as autonomous Glycyl radical cofactors reconstituting the catalytic center of oxygen-fragmented pyruvate formate-lyase. *Biochem. Biophys. Res. Commun.* 285 456–462. 10.1006/bbrc.2001.5186 11444864

[B104] Whelan K. F. Colleran E. Taylor D. E. (1995). Phage inhibition, colicin resistance, and tellurite resistance are encoded by a single cluster of genes on the IncHI2 plasmid R478. *J. Bacteriol.* 177 5016–5027. 10.1128/jb.177.17.5016-5027.1995 7665479 PMC177279

[B105] Whelan K. F. Sherburne R. K. Taylor D. E. (1997). Characterization of a region of the IncHI2 plasmid R478 which protects *Escherichia coli* from toxic effects specified by components of the tellurite, phage, and colicin resistance cluster. *J. Bacteriol.* 179 63–71. 10.1128/jb.179.1.63-71.1997 8981981 PMC178662

[B106] Wojnowska M. Walker D. (2019). FusB energises import across the outer membrane through direct interaction with its ferredoxin substrate. *Microbiology* 11 e2020–e2081. 10.1101/749960 PMC7593965 33109756

[B107] Ye J. Coulouris G. Zaretskaya I. Cutcutache I. Rozen S. Madden T. L. (2012). Primer-BLAST: A tool to design target-specific primers for polymerase chain reaction. *BMC Bioinformatics* 13:134. 10.1186/1471-2105-13-134 22708584 PMC3412702

[B108] Yuan J. Jin F. Glatter T. Sourjik V. (2017). Osmosensing by the bacterial PhoQ/PhoP two-component system. *Proc. Natl. Acad. Sci. U S A.* 114 E10792–E10798. 10.1073/pnas.1717272114 29183977 PMC5740661

[B109] Zanegina O. Kirsanov D. Baulin E. Karyagina A. Alexeevski A. Spirin S. (2016). An updated version of NPIDB includes new classifications of DNA–protein complexes and their families. *Nucleic Acids Res.* 44 D144–D153. 10.1093/nar/gkv1339 26656949 PMC4702928

