# Supplementary material for: The PhoPQ Two-Component System Is the Major Regulator of Cell Surface Properties, Stress Responses and Plant-Derived Substrate Utilisation During Development of Pectobacterium versatile-Host Plant Pathosystems
Source: Front Microbiol. 2021 Jan 15;11:621391. doi: 10.3389/fmicb.2020.621391 (PMC7843439; doi:10.3389/fmicb.2020.621391)
Supplement: Supplementary file 1 [file Table_1.docx]

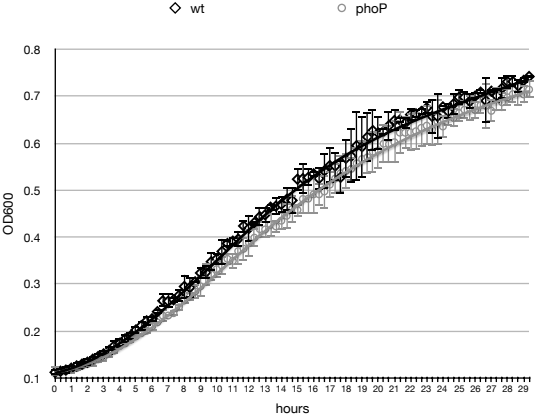


**Supplementary figure 1.** Growth curves of *Pve* strains. Overnight cultures of the wild type (wt) and *phoP* mutant (phoP) *Pve* strains were grown with aeration in MMA at 28 °C to an optical density at 600 nm (OD600) 0.4 and then diluted to OD600=0.1 in the same medium. 100 µl of diluted cultures were grow placed into the wells of a multititer plate and grown at 28 °C for 29 hours with culture mixing and optical dencity measurement performed every 20 minutes by a plate reader. Four separate cultures were used for each strain. Average values with 95% confidence intervals are shown at each time point.


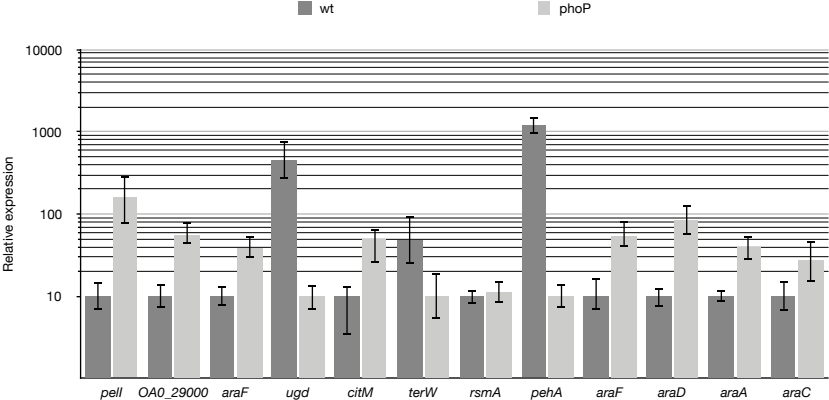


**Supplementary figure 2.** Verification of selected DEGs by qPCR. For qPCR measurement, RNA was isolated from the wild type (wt) or *phoP* mutant *Pve* cells grown in MMA with 0.5% sodium polypectate. Mean values of relative expression levels with 95% confidence intervals are shown.


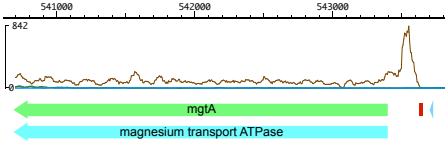


**Supplementary figure 3.** RNA-Seq coverage plot of the *mgtA* gene. Coverage plots are brown for the *phoP* mutant and blue for the wild type strain. Blue triangle – PhoP binding site. Red rectangle – putative Rho-independent terminator of the upstream gene.

**(****A****)**
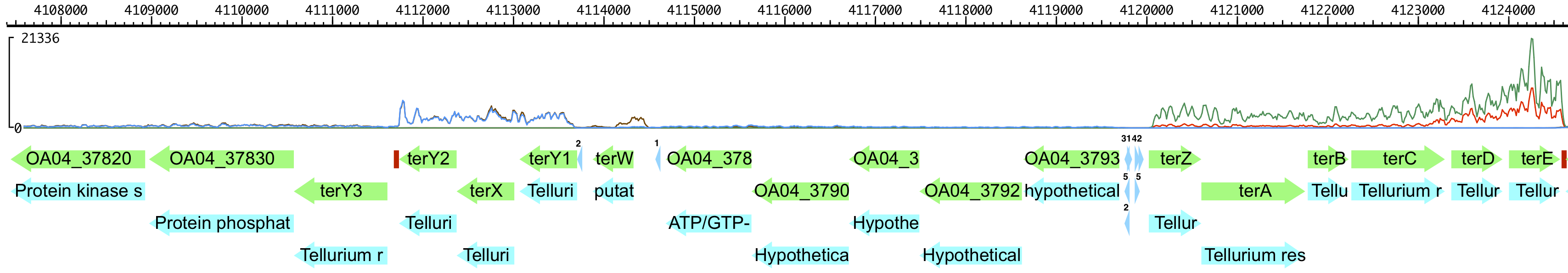


**(****B)**
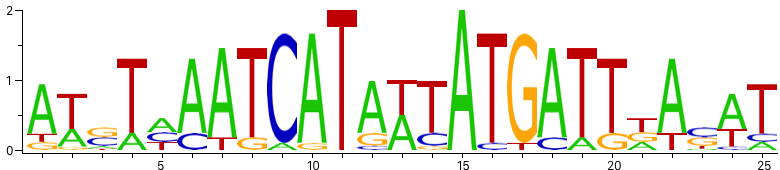


**Supplementary figure 4.** Tellurite resistance locus in *Pve*. **(A)** Gene and regulatory element arrangement. Transcription factor binding sites are shown as blue triangles and labelled as 1 (PhoP), 2 (TerW), 3 (CRP), 4 (SlyA) and 5 (FNR). Transcription terminators are shown as red rectangles. RNA-seq coverage graphs are drawn to the same scale (shown on the left) in green (*phoP*) and red (wild type) for the top strand and blue (*phoP*) and brown (wild type) for the bottom strand. **(B)** Sequence logo of the putative TerW binding site.

**SUPPLEMENTARY TABLES**

**Supplementary table 1.** Synthetic oligonucleotides

| Name | Sequence |
| --- | --- |
| phoPf | 5′-GATGAGCTCTATTCGACAGAGGRTATA-3′ |
| phoPr | 5′-GCCTCTAGAGGTCGCTTCTTATCGCTCAT-3′ |
| pjp1 | 5′-GTAAAACGACGGCCAGT-3′ |
| pjp2 | 5′-CAGGAAACAGCTATGAC-3′ |
| phoPQf1 | 5′-ACCGAGCTCAAACCCGATCGCAATCTCTCT-3′ |
| phoPQr | 5′-ATTTCTAGACAATCAGTATTTTGGCGATAAGT-3′ |
| expIqf | 5′-TGGGCCGTGCAATGTACTGA-3′ |
| expIqr | 5′-CGTCCCGGTAATCATGTTAGGGTAT-3′ |
| citMqf | 5′-CCCATGCTCCCGGCGATTAT-3′ |
| citMqr | 5′-CGCTGCCGTTGCCTAGATTG-3′ |
| OA04_2900qf | 5′-CGCTCCGTATTGCGTAACGT-3′ |
| OA04_2900qr | 5′-GCTCGACCAGTAGGCGGAAA-3′ |
| araFqf | 5′-TCGGTTGGCCTTTAGCGTTCA-3′ |
| araFqr | 5′-CTGTACGCCGGATCCGAAGT-3′ |
| ffhqf | 5′-GTATGGGCGATGTGCTTTCACT-3′ |
| ffhqr | 5′-AAATCAAACCCATCGCCCTTCTT-3′ |
| gyrAf | 5′-AAAGAGACGGTCGATTTTGTGC-3′ |
| gyrAr | 5′-TCAGGTTGTGCGGTGGAATG-3′ |
| citWqf | 5′-CGCAGGCAAACCCGAAGAAT-3′ |
| citWqr | 5′-TTTGTGCTGGCGGGGGTATT-3′ |
| pelIqr | 5′-ACCGCCAATCCACAGTCAGA-3′ |
| pelIqf | 5′-ATGGTGCTGGTGCCGTTGAT-3′ |
| ugdqf | 5′-AACGCTCAATCTCGGCGTCAT-3′ |
| ugdqr | 5′-GCGCAACAGCATCAGGTTGT-3′ |
| terWqf | 5′-CAGTACACGGGTCAACGTAGGT-3′ |
| terWqr | 5′-CGTGTTTTCCAGCTCGCCAAT-3′ |
| phoPqf | 5′-TTACGTGACCAAGCCGTTCCA-3′ |
| phoPqr | 5′-TCACCAGTTCACGACGCGATA-3′ |
| pehAf | 5′-TGTGGCGTGGTCGATACAAA-3′ |
| pehAr | 5′-AGATCCCACCAGCTCACCTT-3′ |
| phoPo1 | 5′-TATGACCATTTCCAGCCTG-3′ |
| phoPo2 | 5′-GAGGGTAAACCGGGTAT-3′ |
| expIo2 | 5′-GACAACGCGAGAAAAACCA-3′ |
| expIo1 | 5′-GTCTCGCGCTTCCGGATT-3′ |
| pehAo1 | 5′-TTTATTGAGGTTAATCAGA-3′ |

**Supplementary table 2.** Plasmids

| Plasmid name | Characteristics | Source/reference |
| --- | --- | --- |
| pJP5603 | Km^R^ Ori_R6K_ *mob* | (Penfold and Pemberton, 1992) |
| pJP5603::'phoP' | pJP5603 with insertion of NdeI-PvuII *phoP* fragment | This work |
| pJQ200KS | Gm^r^ *lacZ*α *mob* oriV_p15A_ | (Quandt and Hynes, 1993) |
| pZH449 | 1.8 kb ClaI-HindIII deletion of pJQ200KS which removed *sacB* | This work |
| pPQ | pZH449::phoPQ | This work |

**Supplementary table 3.** Full list of PhoP-dependent genes of *Pve*

| Gene | locus_tag | Product | FDR | P Value | Fold change |
| --- | --- | --- | --- | --- | --- |
| *visP* | OA04_00090 | virulence- and stress-related protein; Bacterial OB fold (BOF) protein | 7.07E-10 | 1.36E-05 | 2.73 |
| *-* | OA04_01500 | LysR family transcriptional regulator | 6.95E-07 | 3.19E-04 | 0.34 |
| *-* | OA04_02820 | amino acid ABC transporter substrate binding periplasmic protein | 3.16E-03 | 6.93E-03 | 2.89 |
| *scrK* | OA04_04060 | aminoimidazole riboside kinase | 3.61E-03 | 1.20E-01 | 0.45 |
| *nrdD* | OA04_04300 | anaerobic ribonucleoside triphosphate reductase | 2.16E-02 | 1.38E-01 | 2.19 |
| *-* | OA04_04800 | ABC-2 type transporter | 2.21E-06 | 9.32E-05 | 3.54 |
| *-* | OA04_04810 | ABC-type multidrug transport system permease component | 2.69E-07 | 1.95E-05 | 3.55 |
| *-* | OA04_04820 | membrane protein | 1.78E-10 | 3.62E-10 | 6.47 |
| *-* | OA04_04830 | Outer membrane efflux family protein | 1.01E-10 | 4.84E-10 | 5.46 |
| *mgtA* | OA04_05060 | magnesium transport ATPase | 8.38E-15 | 1.32E-15 | 15.41 |
| *-* | OA04_05190 | ABC transporter extracellular solute binding protein | 4.57E-03 | 8.22E-03 | 2.96 |
| *-* | OA04_05580 | putative efflux permease | 3.73E-06 | 1.03E-05 | 4.11 |
| *entC* | OA04_05590 | enterobactin synthetase subunit C (isochorismate synthase) | 5.55E-03 | 4.29E-03 | 4.09 |
| *yjbJ* | OA04_05950 | osmotic stress-induced protein\|RpoS regulon | 3.18E-03 | 6.66E-01 | 2.00 |
| *ubiU* | OA04_06900 | ubiquinone biosynthesis protein | 1.40E-02 | 7.48E-03 | 5.99 |
| *-* | OA04_07070 | coagulation factor 5/8 type domain protein | 1.18E-05 | 3.39E-01 | 2.00 |
| *togT* | OA04_08060 | oligogalacturonide transporter | 5.31E-07 | 2.12E-01 | 2.01 |
| *fusB* | OA04_08790 | TonB-like protein | 7.29E-05 | 4.81E-02 | 2.29 |
| *norV* | OA04_09050 | anaerobic nitric oxide reductase flavorubredoxin | 1.97E-02 | 6.35E-02 | 2.55 |
| *-* | OA04_10330 | Inner membrane protein, TerC-like | 1.62E-09 | 7.91E-03 | 0.45 |
| *-* | OA04_10430 | hypothetical protein | 2.76E-06 | 9.94E-05 | 3.58 |
| *ppdC* | OA04_10440 | prepilin peptidase dependent protein C precursor | 4.08E-05 | 1.58E-04 | 4.64 |
| *-* | OA04_10630 | ABC transporter substrate binding protein | 1.37E-04 | 5.17E-02 | 2.31 |
| *gcvB* | OA04_10700 | GcvB RNA | 2.48E-02 | 4.95E-03 | 2.17 |
| *pelI* | OA04_11360 | Pectate lyase PelI | 1.76E-13 | 2.54E-14 | 0.14 |
| *pehA* | OA04_11370 | endo-polygalacturonase | 1.22E-22 | 9.23E-26 | 117.6 |
| *glnK* | OA04_12080 | nitrogen regulatory protein P-II | 1.20E-04 | 2.11E-01 | 0.49 |
| *-* | OA04_12400 | coagulation factor 5/8 type domain protein | 2.06E-08 | 2.07E-01 | 2.02 |
| *pehN* | OA04_12450 | exo-poly-alpha-D-galacturonosidase | 3.17E-07 | 3.32E-02 | 2.15 |
| *hybO* | OA04_12810 | hydrogenase 2 small subunit | 3.65E-02 | 2.30E-01 | 2.03 |
| *hypB* | OA04_12900 | hydrogenase nickel incorporation protein HypB | 2.78E-02 | 9.53E-02 | 2.39 |
| *hypA* | OA04_12910 | hydrogenase nickel incorporation protein HypA | 1.83E-03 | 8.55E-04 | 6.85 |
| *hydN* | OA04_13040 | electron transport protein HydN | 2.47E-04 | 1.32E-02 | 2.71 |
| *-* | OA04_13340 | RNA polymerase sigma factor | 3.15E-04 | 2.46E-02 | 2.55 |
| *tcp* | OA04_13410 | methyl-accepting chemotaxis protein | 2.34E-05 | 1.71E-03 | 0.39 |
| *mtrB* | OA04_14870 | putative methylthioribose ABC transporter, periplasmic binding protein | 1.94E-02 | 1.48E-01 | 2.16 |
| *fdnG* | OA04_14900 | formate dehydrogenase, nitrate-inducible, major subunit | 5.92E-03 | 2.84E-03 | 5.55 |
| *ftp* | OA04_17930 | FAD:protein FMN transferase | 4.83E-09 | 4.64E-03 | 2.29 |
| *slyB* | OA04_18750 | outer membrane lipoprotein | 2.47E-14 | 2.13E-07 | 2.46 |
| *ccmA* | OA04_18220 | cytochrome c biogenesis protein CcmA | 1.14E-02 | 6.16E-03 | 5.88 |
| *napF* | OA04_18370 | ferredoxin-type protein | 1.06E-03 | 2.62E-03 | 3.94 |
| *pgpB* | OA04_18890 | phosphatidylglycerophosphatase B | 5.11E-17 | 7.97E-17 | 5.59 |
| *araD* | OA04_19040 | L-ribulose-5-phosphate 4-epimerase | 4.09E-07 | 2.61E-08 | 0.08 |
| *-* | OA04_20060 | acyltransferase 3 | 4.63E-15 | 9.33E-12 | 3.18 |
| *pelP* | OA04_21040 | periplasmic pectate lyase | 9.99E-21 | 1.94E-22 | 9.67 |
| *-* | OA04_21050 | KdgM-like porin | 6.62E-12 | 1.04E-12 | 9.12 |
| *-* | OA04_21060 | KdgM-like oligalacturonate-specific porin | 7.25E-16 | 5.08E-17 | 25.51 |
| *ychH* | OA04_21530 | putative inner membrane stress-induced protein | 1.90E-04 | 2.56E-02 | 2.31 |
| *ynfK* | OA04_22140 | putative dithiobiotin synthetase | 4.63E-03 | 2.61E-03 | 5.06 |
| *-* | OA04_22250 | putative inorganic ion transporter | 1.99E-11 | 3.49E-02 | 2.10 |
| *araC* | OA04_22270 | DNA-binding transcriptional regulator AraC | 5.86E-16 | 2.26E-16 | 0.11 |
| *araH* | OA04_22280 | L-arabinose transporter permease | 3.98E-18 | 3.41E-19 | 0.07 |
| *araG* | OA04_22290 | L-arabinose transporter ATP-binding protein | 2.23E-18 | 5.77E-20 | 0.05 |
| *araF* | OA04_22300 | L-arabinose-binding periplasmic protein | 5.37E-17 | 1.34E-18 | 0.06 |
| *araB* | OA04_22310 | L-ribulokinase | 1.17E-19 | 9.97E-22 | 0.02 |
| *araA* | OA04_22320 | L-arabinose isomerase | 7.24E-20 | 3.64E-22 | 0.03 |
| *-* | OA04_23860 | hypothetical protein | 3.15E-03 | 5.34E-03 | 3.76 |
| *pepT* | OA04_23990 | peptidase T | 1.24E-02 | 7.77E-03 | 4.93 |
| *-* | OA04_24930 | Protein kinase-like domain protein | 3.65E-02 | 2.94E-01 | 2.36 |
| *-* | OA04_25030 | Glycine betaine/L-proline ABC transporter, ATPasesubunit | 4.35E-04 | 6.74E-03 | 3.09 |
| *citW* | OA04_25230 | citrate/acetate antiporter | 3.17E-05 | 2.07E-04 | 3.79 |
| *focA* | OA04_25450 | formate transporter | 2.97E-03 | 4.88E-02 | 2.31 |
| *-* | OA04_26190 | TonB-dependent receptor | 5.37E-17 | 1.42E-17 | 9.81 |
| *lpxT* | OA04_26310 | Lipid A 1-diphosphate synthase | 1.41E-13 | 3.08E-12 | 4.33 |
| *-* | OA04_28020 | 2-dehydropantoate 2-reductase | 6.45E-06 | 2.05E-01 | 2.03 |
| *cbl* | OA04_28870 | transcriptional regulator CysB-like protein | 5.92E-03 | 1.77E-02 | 2.71 |
| *fetM* | OA04_28960 | high affinity Fe2+ permease | 2.76E-06 | 7.31E-02 | 2.16 |
| *fdhF_2* | OA04_28980 | Formate dehydrogenase H | 4.14E-02 | 1.11E-01 | 2.36 |
| *citM* | OA04_28990 | CitMHS family citrate/H+ symporter | 2.08E-20 | 2.06E-22 | 0.07 |
| *-* | OA04_29000 | putative transcriptional regulator | 3.15E-18 | 3.20E-19 | 0.09 |
| *-* | OA04_29010 | sodium:dicarboxylate symporter | 1.13E-12 | 2.88E-02 | 0.48 |
| *pnl* | OA04_29050 | pectin lyase | 2.70E-09 | 8.12E-05 | 2.63 |
| *-* | OA04_29260 | Abhydrolase superfamily protein | 2.35E-20 | 1.48E-22 | 67.65 |
| *-* | OA04_29520 | extracellular solute-binding protein | 9.98E-04 | 2.77E-03 | 3.27 |
| *-* | OA04_29530 | putative dehydratase | 9.76E-04 | 7.93E-02 | 2.22 |
| *-* | OA04_29540 | ABC transporter permease | 9.52E-05 | 4.35E-04 | 3.39 |
| *-* | OA04_29750 | putative oxidase, cupin superfamily | 4.78E-02 | 5.80E-02 | 2.91 |
| *arnF* | OA04_31350 | 4-amino-4-deoxy-L-arabinose-phosphoundecaprenol flippase subunit ArnF | 4.61E-08 | 3.64E-08 | 8.86 |
| *arnE* | OA04_31360 | 4-amino-4-deoxy-L-arabinose-phosphoundecaprenol flippase subunit ArnE | 1.30E-04 | 1.32E-04 | 6.39 |
| *arnT* | OA04_31370 | 4-amino-4-deoxy-L-arabinose transferase | 2.99E-12 | 1.34E-12 | 11.64 |
| *arnD* | OA04_31380 | Undecaprenyl phosphate-aminoarabinose deformylase | 1.16E-15 | 1.14E-16 | 24.77 |
| *arnA* | OA04_31390 | NAD-dependent UDP-glucuronate dehydrogenase and UDP-ara4N formyltransferase | 1.89E-15 | 7.58E-17 | 33.64 |
| *arnC* | OA04_31400 | undecaprenyl phosphate 4-deoxy-4-formamido-L-arabinose transferase | 1.62E-11 | 9.61E-13 | 28.45 |
| *arnB* | OA04_31410 | UDP-4-amino-4-deoxy-L-arabinose--oxoglutarate aminotransferase | 2.47E-16 | 7.23E-18 | 78.53 |
| *ugd* | OA04_31420 | UDP-glucose 6-dehydrogenase | 3.74E-15 | 1.16E-16 | 49.63 |
| *-* | OA04_32550 | exported choloylglycine hydrolase | 2.47E-14 | 1.76E-10 | 3.02 |
| *-* | OA04_32560 | coagulation factor 5/8 type domain protein | 2.77E-08 | 1.55E-01 | 2.04 |
| *yiuA* | OA04_33210 | ferric-enterobactin ABC-transporter, substrate binding periplasmic protein | 1.45E-18 | 1.79E-19 | 8.36 |
| *yiuB* | OA04_33220 | ferric-enterobactin ABC-transporter, permease component | 1.22E-17 | 2.13E-17 | 5.19 |
| *yiuC* | OA04_33230 | ferric-enterobactin ABC transporter ATP-binding protein | 8.81E-14 | 7.19E-10 | 2.99 |
| *grcA* | OA04_33700 | stress-induced alternative pyruvate formate-lyase subunit | 1.95E-03 | 6.25E-04 | 6.06 |
| *raiA* | OA04_34460 | stationary phase translation inhibitor and ribosome stability factor | 1.72E-02 | 1.36E-01 | 2.18 |
| *gudX* | OA04_36650 | glucarate dehydratase | 5.42E-03 | 3.65E-03 | 4.20 |
| *terW* | OA04_37880 | putative transcriptional regulator of terZABCDE operon | 4.38E-15 | 4.54E-15 | 6.92 |
| *-* | OA04_37930 | hypothetical protein | 2.22E-09 | 7.89E-03 | 0.45 |
| *terZ* | OA04_37940 | Tellurium resistance protein, TerZ | 2.16E-09 | 4.15E-10 | 0.16 |
| *terA* | OA04_37950 | Tellurium resistance protein TerA | 3.15E-18 | 2.03E-18 | 0.16 |
| *terB* | OA04_37960 | Tellurium resistance protein TerB | 2.06E-08 | 8.25E-09 | 0.20 |
| *terC* | OA04_37970 | Tellurium resistance protein TerC | 3.17E-17 | 9.86E-17 | 0.21 |
| *terD* | OA04_37980 | Tellurium resistance protein TerD | 7.26E-10 | 3.13E-03 | 0.45 |
| *terE* | OA04_37990 | Tellurium resistance protein TerE | 6.62E-12 | 8.85E-05 | 0.43 |
| *-* | OA04_38360 | Sugar (Glycoside-Pentoside-Hexuronide) transporter | 7.49E-13 | 4.22E-12 | 0.19 |
| *-* | OA04_38370 | putative alpha-N-arabinofuranosidase | 3.63E-09 | 8.53E-04 | 0.41 |
| *alaE* | OA04_38680 | L-alanine exporter | 1.08E-05 | 2.53E-03 | 2.79 |
| *-* | OA04_40040 | putative efflux permease, MFS superfamily | 7.87E-09 | 3.54E-02 | 2.15 |
| *glpA* | OA04_42210 | sn-glycerol-3-phosphate dehydrogenase subunit A | 1.26E-02 | 1.71E-02 | 3.14 |
| *-* | OA04_42530 | extracellular solute-binding protein family 3 | 1.75E-04 | 9.05E-02 | 2.20 |
| *ytfT* | OA04_43110 | ABC transporter permease | 1.46E-04 | 4.74E-02 | 0.43 |
| *ytfR* | OA04_43120 | galactofuranose ABC transporter ATP-binding protein | 1.02E-11 | 2.81E-11 | 0.17 |
| *ytfQ* | OA04_43130 | galactofuranose/arabinofuranose ABC transporter periplasmic binding protein | 1.18E-10 | 3.12E-11 | 0.14 |
| *metF* | OA04_43310 | 5,10-methylenetetrahydrofolate reductase | 5.24E-03 | 4.59E-03 | 3.58 |
| *sftR* | OA04_43750 | LysR family transcriptional regulator SftR | 7.50E-07 | 1.30E-02 | 2.34 |
| *aegA* | OA04_45430 | oxidoreductase Fe-S binding subunit | 4.29E-03 | 2.12E-03 | 5.43 |
| *-* | OA04_45440 | electron transport protein | 6.54E-03 | 9.70E-02 | 2.31 |
| *-* | OA04_45500 | extracellular solute-binding protein | 8.24E-03 | 5.11E-03 | 4.60 |

**R****efer****en****ces**

Penfold, R. J., and Pemberton, J. M. (1992). An improved suicide vector for construction of chromosomal insertion mutations in bacteria. *Gene* 118, 145–6. doi:1511879.

Quandt, J., and Hynes, M. F. (1993). Versatile suicide vectors which allow direct selection for gene replacement in gram-negative bacteria. *Gene* 127, 15–21.
